# Metabolic reprogramming and Notch activity distinguish between non-small cell lung cancer subtypes

**DOI:** 10.1038/s41416-019-0464-z

**Published:** 2019-05-22

**Authors:** Katherine Sellers, Thaddeus D. Allen, Michael Bousamra, JinLian Tan, Andrés Méndez-Lucas, Wei Lin, Nourdine Bah, Yelena Chernyavskaya, James I. MacRae, Richard M. Higashi, Andrew N. Lane, Teresa W.-M. Fan, Mariia O. Yuneva

**Affiliations:** 1 The Francis Crick Institute, London, UK; 2 G.W. Hooper Research Foundation, University of California, San Francisco, CA USA; 3 Tradewind BioScience, Inc., Daly City, CA USA; 4 James Graham Brown Cancer Center, University of Louisville, Louisville, KY USA; 5 Present Address: Baptist Health Medical Group, Thoracic Surgery, New Albany, IN USA; 6 Present Address: Center for Environmental and Systems Biochemistry, Dept. Toxicology and Cancer Biology, University of Kentucky, Lexington, KY USA; 7 Present Address: Agios Pharmaceuticals, Cambridge, MA USA; 8 Present Address: Dental-Oral Immunity & Infectious Diseases, Dept. Medicine, University of Louisville, Louisville, KY USA

**Keywords:** Cancer metabolism, Non-small-cell lung cancer

## Abstract

**Background:**

Previous studies suggested that the metabolism is differently reprogrammed in the major subtypes of non-small cell lung cancer (NSCLC), squamous cell carcinomas (SCC) and adenocarcinomas (AdC). However, a comprehensive analysis of this differential metabolic reprogramming is lacking.

**Methods:**

Publicly available gene expression data from human lung cancer samples and cell lines were analysed. Stable isotope resolved metabolomics were performed on SCC and ADC tumours in human patients and in freshly resected tumour slices.

**Results:**

Analysis of multiple transcriptomics data from human samples identified a SCC-distinguishing enzyme gene signature. SCC tumours from patients infused with [U-^13^C]-glucose and SCC tissue slices incubated with stable isotope tracers demonstrated differential glucose and glutamine catabolism compared to AdCs or non-cancerous lung, confirming increased activity through pathways defined by the SCC metabolic gene signature. Furthermore, the upregulation of Notch target genes was a distinguishing feature of SCCs, which correlated with the metabolic signature. Notch and MYC-driven murine lung tumours recapitulated the SCC-distinguishing metabolic reprogramming. However, the differences between SCCs and AdCs disappear in established cell lines in 2D culture.

**Conclusions:**

Our data emphasise the importance of studying lung cancer metabolism in vivo. They also highlight potential targets for therapeutic intervention in SCC patients including differentially expressed enzymes that catalyse reactions in glycolysis, glutamine catabolism, serine, nucleotide and glutathione biosynthesis.

## Background

Lung cancer kills more than 1.5 million patients worldwide each year.^1^ Roughly 85% of all lung malignancies are non-small cell lung cancers (NSCLC), which mostly consist of adenocarcinoma (AdC) and squamous cell carcinoma (SCC) histotypes.^2^ Although there are several targeted lung cancer treatments for AdC including the EGFR TKI inhibitors, inhibitors of abnormal ALK and several antibodies among others,^3^ only a few have recently been approved for SCC, including a monoclonal antibody against EGFR, Necitumumab^4^ and immune checkpoint inhibitors Nivolumab^5^ and Pembrolizumab.^6^

Tumour metabolism is an emerging source of novel targets, as cancers alter their metabolism in order to facilitate proliferation, migration and survival in the tumour microenvironment.^7^ The notion that altered metabolism is a hallmark of tumorigenesis^8^ is underscored by the discovery that driver oncogenes and tumour suppressors modulate enzyme expression and/or post-translational modifications, which leads to specific metabolic changes.^9^ Importantly, these oncogene-driven metabolic changes create cancer cell dependencies that can be exploited therapeutically.^10,11^

We, and others, have shown that human and mouse NSCLC’s have a distinct metabolic profile and activity compared with non-cancerous (NC) lung tissue.^12–15^ [U-^13^C]-glucose infusion into human NSCLC patients and stable isotope-resolved metabolomic (SIRM) analysis demonstrated that tumours increase oxidation of glucose through glycolysis and the Krebs cycle.^13,14^ However, these studies assessed glucose utilisation in NSCLC tumour tissues without addressing how metabolism in tumours of different histotypes is altered in comparison with normal lung (metabolic reprogramming). Previous metabolomic and proteomic studies of human and murine lung tumours have suggested that AdCs and SCCs differ metabolically,^16,17^ but none robustly defined which metabolic pathways were altered specifically in SCCs.

Therefore, we asked whether the metabolic reprogramming observed in human NSCLC is histology-specific. Transcriptomics revealed a 24-metabolic gene signature, which distinguished SCCs and AdCs and which was consistent with alterations in 5 metabolic pathways as assessed by SIRM in lung cancer patients and tissue slices. While conducting our bioinformatic analysis we discovered that the upregulation of Notch target genes was a distinguishing feature of SCCs. To investigate this further, we utilised a murine model of *NOTCH1*-driven lung cancer. Overexpression of activated *NOTCH1* and its downstream target *MYC* in mouse lung produced tumours that recapitulated the SCC-distinguishing metabolism. Interestingly, the relationship between histotypes, oncogenic signalling and metabolic gene signature were lost in established cancer cell lines.

Together, this study expands on previous research by defining histotype-specific metabolic reprogramming in NSCLCs and monitoring carbon utilisation from isotopically labelled glucose and glutamine into pathways beyond glycolysis and the Krebs cycle. Moreover, it links metabolic reprogramming to oncogenic signalling by demonstrating a Notch-associated metabolic phenotype in lung SCCs, which could represent novel vulnerabilities for future therapeutic intervention. Finally, it demonstrates the importance of using in vivo systems to evaluate the metabolic remodelling in different tumour types.

## Material and methods

### Human gene expression analysis

Oncomine^TM^ (Compendia Bioscience) was used to extract the top 5% upregulated genes in SCC from four databases.^18–21^ Twenty eight AdC and 58 SCC samples were analysed by Zhu et al.^18^; 65 normal lung samples, 45 AdCs and 27 SCCs were analysed by Hou et al.^19^; 30 AdCs and 155 SCCs were analysed from TCGA dataset^20^, and 127 AdCs and 21 SCCs were analysed by Bhattacharjee et al.^21^ Genes that overlapped in at least three of the four databases were analysed by Panther Pathway Analysis.^22^ Hierarchical clustering or principal component analysis (PCA) was performed on microarrays from the Hou database^19^ using Cluster (Michael Eisen of UC Berkley) and Java Treeview^23^ or SimcaP (MKS Data Analytics), respectively. Central carbon metabolism enzymes were filtered based on the gene having a GO Molecular Function of catalytic activity and a GO Biological Process term related to sugar, amino acid, nucleotide or energy metabolism. To determine whether Notch was active in a tissue, a thresholding method was employed. Gene expression was normalised to the median expression in the normal lung tissue. Notch was considered active if 4 of 5 Notch targets (*HES1, HES2, HEY1, HEY2* and *NRARP*)^24^ were overexpressed by 1SD of the NC’s median. This gave an *α* = 0.046 for the Notch signature. Heat maps were exported from Oncomine^TM^. KM plots were generated using Kaplan–Meier Plotter.^25^ JetSet best probes were used for the 24 SCC metabolic signature genes or the Notch targets above and patients were split based on expression in the upper tertile.

Additionally, AdC and SCC patient RNA-seq gene expression data were downloaded from the Genomic Data Commons (GDC) repository using the TCGA biolinks R/Bioconductor package.^26^ Only the harmonised data were selected. The data set contained 59 normal tissue samples from AdC patients and 49 from SCC patients, 535 AdC and 502 SCC samples. Associated clinical data were also downloaded from the GDC repository. Three read counts matrices were build using the AdC and SCC samples. Read counts were normalised and transformed using variance-stabilising transformation (VST) using the DESeq2 R/Bioconductor package.^27^ VST counts matrix was filtered with the 25 genes signature (in contrast to microarray data the expression of CKTM1A and CKMT1B genes was quantified separately). *Z*-scores were computed, which were used to generate the heatmaps using the ComplexHeatmap R/Bioconductor package^28^ with the clustering default parameters. Principal Component Analyses (PCA) was performed on the VST counts matrix for the 25 genes signature. The first 10 principal components were used to perform ANOVA of a tumour stage. The Benjamini–Hochberg procedure^29^ was applied on the ANOVA *p* values to control the FDR.

### Human tissue SIRM

Lung cancer patients with suspected primary lung cancer but without diagnosed diabetes were recruited on the basis of surgical eligibility according to an IRB-approved protocol as previously described.^30^ Patients were overnight-fasted (>8 h) and then randomly grouped into two cohorts. In one of the cohorts, patients were administered 10 g [U-^13^C]-glucose intravenously and preoperatively 2.8 ± 0.5 h prior to VATS wedge resection. Another cohort did not receive a glucose injection. The extent of resection was determined by the surgeon according to clinical criteria. Most of the specimens were obtained from wedge resections to minimise surgical times while the remainder was acquired in <5 min after the pulmonary vein was clamped; both practices helped avoid development of significant ischaemia in resected tissues. Immediately after resection, the tumour was transected and sections of cancerous and surrounding NC lung tissue at least 2 cm away from the tumour were biochemically quenched by flash freezing in liquid nitrogen. The margins of the tumour were initially assessed by the surgeon via visual inspection. Parallel tissue samples were sent to on-site pathologists for confirmation of diagnosis and cancer-free margin. The remaining specimen was preserved in buffered formalin for detailed pathological examination.

The tissues from both groups of patients were used for metabolomics analysis. Additionally, some tissues from both groups were thin sliced using a Weck microtome (0.5–1 mm thick, 0.5–1 cm^2^ area) in the operating room. These slices were immediately placed in T25-flasks containing 8 mL DMEM with the appropriate tracer (either 10 mM ^13^C-glucose, 2 mM glutamine or 10 mM glucose and 2 mM [U-^13^C,U-^15^N]-glutamine) and 10% dialysed foetal bovine serum (FBS), and then transferred to a CO_2_ incubator set to 37 °C and 5% CO_2_ as previously described.^31^ The flasks were continuously rocked for 24 h for aeration and to maintain constant nutrient supplies at the tissue surface, while avoiding local build-up of waste products such as lactate. The slices were then washed in cold PBS and frozen in liquid nitrogen.

The frozen tissue samples were pulverised to <10 µm particle size in liquid nitrogen using a freezer mill (Spex). Metabolites were extracted from the ground powder with a 2:1.5:1 ratio of acetonitrile:water:chloroform giving a total volume of 4.5 mL followed by centrifugation for 20 min at 4000 × *g* at 4 °C. This afforded a two-phase partition, separated by insoluble protein residue. The polar phase was aliquoted and lyophilised for analysis by NMR and GC-MS. The NMR fraction was dissolved in 350 μL 1-mM perdeuterated EDTA in D_2_O containing 30 nmol d_6_-DSS. The GC-MS fraction was dissolved in 50 μL water containing 50 nmol norleucine internal standard and acidified with 33 μL 40% trichloroacetic acid (TCA) to a final 10% TCA concentration at 4 °C. The sample was re-lyophilised, then brought up in 50 μL 1:1 acetonitrile:*N*-(t-butyldimethylsilyl)-*N*-methyltrifluoroacetamide (MTBSTFA, Campbell Science) and sonicated for 3 h, followed by overnight incubation at RT. The protein residues were extracted by homogenisation in a 2% sodium dodecyl sulphate (SDS), 62.5 mM Tris, and 1 mM DTT, pH 6.8 buffer for protein determination using the Pierce BCA method (ThermoFisher Scientific).

Media samples were deproteinise using acetone precipitation, followed by lyophilisation and dissolution in D_2_O containing DSS-d_6_. NMR spectra were acquired at 20 °C on a Varian Inova 14.1T spectrometer (Varian, Inc.) equipped with a 5 mm HCN cold probe (tissue extracts) or on an Agilent DD2 14.1T spectrometer equipped with a 3 mm HCN cold probe (media extracts). 1D proton spectra were recorded with presaturation of the solvent resonance, an acquisition time of 2 s, and a recycle time of 5 s. 1D ^1^H-^13^C HSQC spectra were recorded with acquisition times of 0.15 s in *t*_2_. Spectral analysis was performed with MestReNova software (Mestrelab Research). Spectra were zero filled to 128K points (presat) or 8k points (1D HSQC) and apodized with an unshifted Gaussian function and 1 (^1^H NMR) or 6 Hz (1D ^1^H-^13^C HSQC) line broadening exponential. Chemical shifts were referenced to DSS at 0 ppm. After manual phase and baseline correction, peaks were integrated using the global deconvolution method of the software. Metabolites were identified based on chemical shifts as previously determined using 2D methodologies and comparisons to standard.^29,30^ Metabolites were quantified using the –Si-(CH_3_)_3_ DSS peak at 0 ppm (for ^1^H-NMR) or the 3-^13^C lactate peak at 1.32 ppm (for ^1^H-^13^C HSQC) as a reference.

GC-MS samples were analysed on a ThermoFinnigan PolarisQ GC-Ion trap MSn system (ThermoFisher Scientific). One microliter of sample injected (inlet 280 °C) onto a 5% phenyl capillary column (50 m × 0.15 mm × 0.25 μm (SGE Forte, Victoria, Australia)) using 1.5 mL/min helium as carrier gas. An oven temperature of 60 °C was held for 2 min, followed by gradients to 150 °C (20 °C/min) and then 300 °C (6 °C/min). The transfer line was held at 280 °C. The MS was operated in segment scan mode (scan ranges: 140–206, 209–280 and 283–650 *m*/*z*, scan-rate 1 scan per 0.97 sec. Metabolites were identified and quantified by comparison to the retention times, fragmentation patterns, and peak areas of authentic standards. Ion peaks were integrated using Xcalibur software (ThermoFisher Scientific). Quantifications were normalised to the internal standard norleucine.

### N1ICD and MYC overexpression

The description of the transgenic mouse models used in this study has been previously published.^24,32^ Briefly, mice expressing rtTA under the control of the rat CCSP promoter were crossed with mice encoding *N1ICD* and *MYC* under the transcriptional control of a tetracycline-response element. The CCSP promoter was used to achieve lung-specific expression and mainly led to activation of *N1ICD* and *MYC* in the distal epithelium, presumably type 2 pneumocytes, as opposed to the Club cells of the bronchial epithelium.^24^ As previously reported by Allen and colleagues, the co-expression of *MYC* and *N1ICD* cooperates to produce papillary adenocarcinoma (PA) tumours within the alveolar epithelium.

### Mouse tissue SIRM

Control and tumour-bearing male mice were given a bolus injection of 20 mg [U-^13^C]-glucose or 2 × 7.2 mg [U-^13^C]-glutamine via tail vein 15 min prior to dissection as described previously.^11^ Lung tumours and normal lung were dissected and flash frozen in liquid nitrogen. Tissues were processed for NMR analysis as described for humans. For GC-MS analysis, metabolites from ~1 to 5 mg of tissue were extracted with 400 μL ice-cold chloroform and 200 μL methanol. Samples were vortexed for 30 s and metabolites extracted by pulse sonication for 1 h. Samples were centrifuged for 30 min at 21,000 × *g* and 4 °C. The supernatant was collected, and the tissue pellet was resuspended in 400 μL methanol and 200 μL water containing 5 nmol norleucine. Metabolites were re-extracted as above, and the two supernatants were combined and dried. Metabolites were phase-partitioned using 3:3:1 methanol:water:chloroform (v/v) and subsequent centrifugation (21,000 × *g*, 4 °C, 30 min). Polar (upper) and apolar (lower) phases were separated by an interphase of insoluble protein.

Polar metabolites were dried and washed twice in methanol, followed by derivatizing overnight (RT) by methoxymation (20 mg/mL methoxyamine-hydrochloride in pyridine (both Sigma-Aldrich)) and subsequent trimethylsilylation (99:1 BSTFA+TMS (Supelco Analytical)) for more than 1 h, before injection onto the GC-MS. Proteins were extracted from the insoluble residue by homogenisation in a 2% SDS, 62.5 mM Tris, and 1 mM DTT, pH 6.8 buffer for protein determination using the Pierce BCA method (Thermo Fisher Scientific).

Polar metabolite extracts were analysed by GC-MS (Agilent 7890A-5975C), as previously described.^33^ Identification, abundance and label incorporation of individual metabolites was estimated as previously described.^34^

### Percent enrichment calculations

The level of enrichment of individual isotopologues (m+x) of metabolites was estimated as the percentage of the metabolite pool containing x ^13^C atoms after correction for natural abundance:

$${\mathrm{Enrichment}} \, \, {\mathrm{of}} \, \, {\mathrm{m}} + {\mathrm{x}} = \frac{{{\mathrm{Cm}} + {\mathrm{x}}}} {{{\sum} {{\mathrm{Cm}} + 0 + {\mathrm{Cm + 1}} \ldots {{ +\, {\mathrm{Cm}} + {{\mathrm{i}}}}} }}}{\times 100\% }$$

% carbons enriched for a metabolite with i isotopologues was calculated by:

$$^{13}{\mathrm{Cmet}} = \frac{{m + 1}}{i} + 2\frac{{m + 2}}{i} \ldots + {\mathrm{i}}\frac{{m + i}}{i}$$

### qPCR

RNA was isolated from frozen, ground lung and tumour tissues with TRI Reagent (Sigma). Genomic DNA with degraded with DNA-free kit (Ambion, Life Technologies). Total RNA was measured, and cDNA was generated following the manufacture’s protocol for the High-Capacity cDNA Reverse Transcription Kit (ThermoFisher Scientific) and using 25 μg of RNA in a final volume of 50 μL. RT qPCR reactions were carried out for all genes of interest using TaqMan Gene Expression Assays (ThermoFisher Scientific) on a ViiA 7 RT PCR System (ThermoFisher Scientific). In each 20 μL TaqMan reaction, 1 μL cDNA (corresponding to 50 ng RNA) was mixed with 1 μL TaqMan Gene Expression Assay and 10 μL Taqman Universal PCR Master Mix (Applied Biosystems) and 8 μL water. All reactions were run in duplicate and C_t_ values for the genes of interest were normalised to the reference gene 18s.

### Electrophoresis and western blotting

Proteins were extracted from the frozen tissue or cells harvested via trypsinization by homogenisation in a 2% SDS and 62.5 mM Tris and were heat-denatured at 90 °C for 10 min, then centrifuged at 21,000 × *g* for 30 min at 4 °C. Protein extracts were mixed with a loading buffer give a final concentration of 10% glycerol, 2% SDS, 60 mM Tris pH 6.8, 0.01% bromophenol blue and 10% β-mercaptoethanol. Approximately 20 µg protein per sample was then loaded on a 10% Acrylamide SDS-PAGE gel with a 6% stacking gel and subjected to electrophoresis for ~2 h at 80V in 0.3% Trizma Base, 1.44% glycine and 0.1% SDS. The separated proteins were transferred to a nitrocellulose membrane (BioRad) by blotting at 400 mA for 2 h at 4 °C in 0.3% Trizma Base, 1.44% Glycine, 0.005% SDS and 20% methanol.

Protein loading was assessed by incubating the blot in 0.1% (w/v) Ponceau S red in 5% acetic acid for 5 min, followed by rinsing in water. The blot was blocked for 1 h at RT in 5% powdered milk in phosphate buffered saline plus 0.1% Tween-20 (PBST) and incubated overnight at 4 °C in an appropriate dilution of the primary antibody:

MYC (1:1000, 1472-1, Epitomics)

Notch (1:500, ab4990, Abcam)

HES1 (1:750, ab71559, Abcam)

GAPDH (1:5000, 2118s, Cell Signaling)

TPI (1:500, ab135532, Abcam)

PSAT (1:500, 20180-1-AP, Protein Tech)

PHGDH (1:1000, sc292792, Santa Cruz)

ME1 (1:1000, ab97445, Abcam)

ME2 (1:500, ab139686, Abcam)

GOT1 (1:250, ap2947a, Abgent)

GOT2 (1:500, ab93928, Abcam)

IDH1 (1:1000, 12332-1-AP, Protein Tech)

IDH2 (1:2000, ab55271, Abcam)

ASNS (1:500, 1732, Epitomics)

Actin (1:50,0000, A2228, Sigma)

After rinsing, the membranes were incubated for 1 h at RT in a 1:5000 dilution of an appropriate secondary antibody, e.g. horseradish peroxidase (HRP) conjugated goat anti-mouse or anti-rabbit IgG (Santa Cruz). Finally, after incubation with chemiluminescent HRP substrate (Amersham ECL Prime Western Blotting Detection Reagent, GE Healthcare).

### Statistical analysis

Significance in gene expression for the human microarray data was determined using one-way ANOVA with Tukey correction for multiple comparisons between tissue groups, followed by Bonferroni correction for multiple comparisons of different genes. For the human metabolic data, AdC and SCC tumours were compared to their paired NC counterparts using the paired Student’s *t*-test. To test for significance between AdC and SCC, the Welch *t*-test was performed on the log-ratio of the tumour to matched normal. For mouse gene expression, significance was determined by Welch’s *t*-test. The Bonferroni correction for multiple comparisons of different genes was not conducted because the analysis was restricted to planned comparisons based on genes found to be significant from the human microarray. For the mouse metabolic data, *MYC*+*N1ICD* tumours were compared to NC lung from age-matched littermates using the Welch *t*-test. All statistical tests were two-tailed. All statistical tests were calculated using Prism (GraphPad).

## Results

### Human lung SCCs and AdCs have distinct metabolic gene expression profiles

In order to determine which biological processes distinguish SCCs from NC lung tissues and lung carcinomas of different histotypes, we used Oncomine^TM^ to extract the top 5% genes upregulated in SCCs in comparison with NC tissues and/or AdCs from four databases^18–21^ (Fig. S1a). Genes that overlapped in at least three of the four databases were analysed by PANTHER Gene Ontology.^22^ Genes encoding proteins with enzymatic activity, including metabolic enzymes, constituted the bulk of overlapping genes (Fig. S1b, Table S1).

**Fig. 1:**
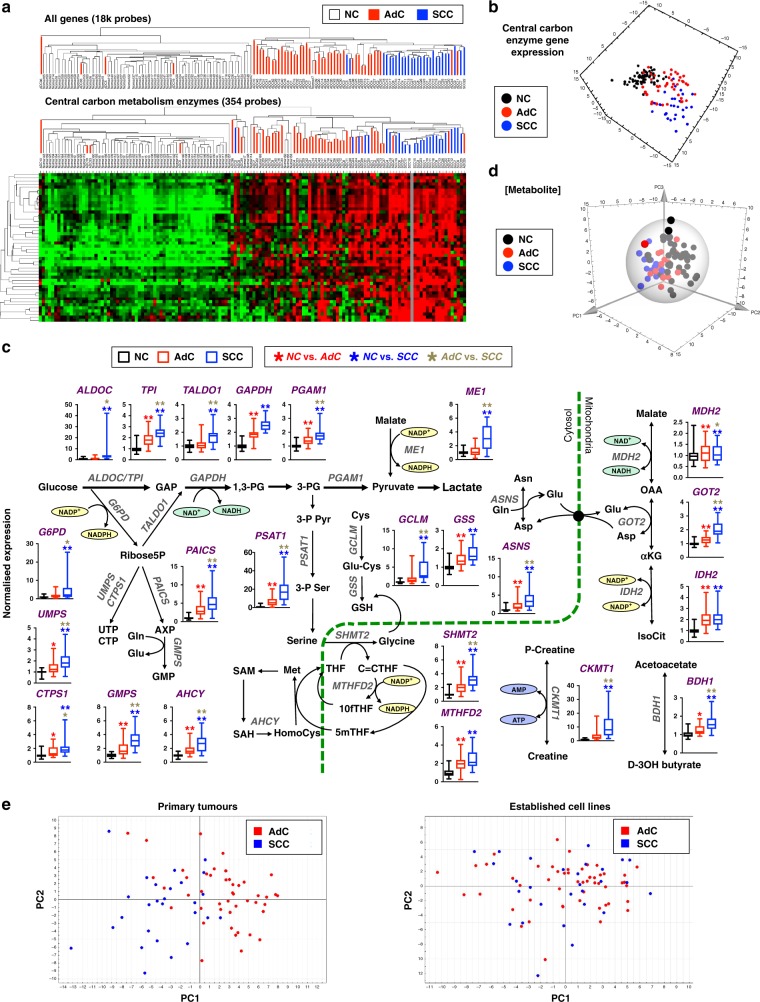
Non-cancerous lung tissue (NC), adenocarcinomas (AdC) and squamous cell carcinomas (SCC) have distinct metabolic phenotypes. **a**: Hierarchical clustering of gene expression from AdC, SCC and NC lung using 18,000+probes or only probes for 354 enzymes in central carbon metabolism from the Hou database^19^ (see Fig. S2 for full heat map). **b** PCA of enzyme expression from the Hou database^19^ R2X[1] = 22%, R2X[2] = 8% and R2X[3] = 5%. **c** 23/24 genes in the SCC-specific metabolic signature mapped onto their interconnecting pathways. Relative expressions are shown as box and whisker plots for AdCs (*n* = 45) and SCCs (*n* = 27), normalised to the median expression in NC tissues (*n* = 65). Whiskers represent min to max. One-way ANOVA with Tukey correction for multiple comparisons between tissue groups and Bonferroni correction for multiple comparisons of different genes. *P < 0.05; **P value < 0.01. See Table S2 for *P*-values, *F* values and degrees of freedom. **d** PCA performed on paired NC and tumours resected from human patients (*n* = 31) based on 55 metabolites measured by GC-MS and NMR R2X[1] = 33.2%, R2X[2] = 13.6% and R2X[3] = 7.5%. **e** PCA of the expression of enzymes in primary tumours (Hou database, top panel, R2X[1] = 12%, R2X[2] = 8%) and commercial cell lines (Cancer Cell Line Encyclopedia from the Broad Institute, bottom panel, R2X[1] = 8%, R2X[2] = 7%). See also Fig. S1–S3 and Table S2

We then asked whether the expression of enzymes that participate in central carbon metabolism alone was sufficient to distinguish SCCs from NC lung and AdCs. Hierarchical Clustering (Fig. 1a, Fig. S2a) and PCA (Fig. 1b, Fig. S2a) were performed on samples from the Hou et al. database^19^ because it contained a large cohort of NC lung samples and allowed us to compare the metabolic reprogramming in different NSCLC histotypes relative to benign lung. We used only those genes that encode enzymes of amino acid, carbohydrate, nucleotide, energy or one-carbon metabolism. Hierarchical clustering readily segregated normal from cancer tissues. The tumour cluster contained a sub-cluster of SCC tumours (Fig. 1a). Accordingly, by PCA, cancerous tissues were well resolved from NC lung in the first principal component (PC1) and SCCs were resolved from AdCs in PC3 (Fig. S2a). Twenty-four genes were (1) responsible for the separation of SCCs in PCs 1 and 3 by PCA as determined by the loadings plot; (2) were found within the two gene clusters revealed by hierarchical clustering with higher expression in SCC tumours than in NC and AdC and (3) were statistically overexpressed in SCC relative to NC. Twenty of these 24 genes were also statistically overexpressed in SCC relative to AdC tissues after Bonferroni correction (Fig. 1c). The differential pattern of changes in the expression of these genes between AdC and SCC tumours relative to NC lungs suggests metabolic reprogramming in NSCLC is histology specific.

We verified the increased expression of the 24-metabolic gene signature in SCCs relative to AdCs or NC and AdCs (where the gene expression data for NC samples were available) in additional databases (Fig. S2a) and TCGA RNA-seq data set (Fig. S3a). Figure 1c shows how 23 of the 24 genes map in the metabolic network. These are genes for enzymes involved in glycolysis (ALDOC, GAPDH, TPI and PGAM), the pentose phosphate pathway (G6PDH, TALDO1), nucleotide biosynthesis (CTPS1, GMPS, UMPS and PAICS), serine biosynthesis/one carbon metabolism (PSAT1, SHMT2, MTHFD2 and AHCY), glutathione biosynthesis (GCLM, GGH and GSS), the malate-aspartate shuttle/Krebs cycle (GOT2, IDH2, MDH2 and ME1), creatine metabolism (CKMT1), butyrate metabolism (BDH1) and asparagine metabolism (ASNS). Together these data show that primary SCC and AdC tumours reprogramme metabolic gene expression differently. Importantly, the 24-gene signature correlates with the stage of the disease (Fig. S3b) and high expression of this 24-gene signature is predictive of a worse overall survival in NSCLC and SCC patients (Fig. S3c).

Given the different expression of metabolic genes, we then sought to determine whether this was associated with changes in the SCC metabolome. We analysed paired cancer and non-cancerous tissues from 39 patients with resectable tumours. Metabolites were measured by gas-chromatography mass spectrometry (GC-MS) and nuclear magnetic resonance (NMR) spectroscopy. PCA was performed using 55 identified and quantified metabolites from matched pairs of tissues. PCA was able to separate lung tumours from NC lungs (Fig. 1d). Furthermore, pairs of tumour and adjacent benign lung tissues from the same patient did not cluster together. This indicates that the changes in metabolism associated with NSCLC tumorigenesis are more distinct than the metabolic differences between individuals due to age, sex, race, genetic make-up or environmental factors such as smoking history.

Similar to the PCA based on metabolic gene expression (Fig. 1b), the PCA based on metabolites (Fig. 1d) suggest that SCC tumours have a more distinct metabolic phenotype from NC tissues than AdC. We also performed PCA on the expression of genes encoding metabolic enzymes used above for primary AdCs and SCCs from Hou database^19^ and commercially available cell lines established from NSCLC patients from the Broad Institute Cancer Cell Line Encyclopedia.^35^ Unlike the primary tumours, which separated in PC1 based on histotype, in vitro cultured cell lines did not (Fig. 1e). These results suggest that cell lines no longer display the metabolic gene signature of their parent histotypes. Indeed, of the 21 genes with available data in the Broad-Novartis Cancer Cell Line Encyclopedia, only BDH1 was significantly elevated in SCC cell lines relative to ADC cell lines (Fig. S2b).

Together these data show that primary SCC and AdC tumours have a distinct reprogramming of metabolic gene expression that is not preserved in established cells in culture.

Although mRNA expression provides important information about the regulation of metabolic pathways, it does not necessarily reflect enzymatic activity, which also depends on protein expression and post-translational modifications or allosteric regulation.^35–37^ Although total levels of metabolites can provide a snapshot of a metabolic state of a cell, they by themselves are not sufficient to infer about the activity of a metabolic pathway. Ultimately metabolic activity provides the evidence for whether the changes in genes or protein expression result in changed metabolism. To that end, we employed stable isotope tracers coupled with SIRM to assess the activity of several pathways implicated in the SCC-distinguishing metabolic signature.

### Human lung SCCs display enhanced activity in pathways related to the metabolic gene signature

Four of the 10 glycolytic enzymes were in the SCC-distinguishing metabolic gene signature (Fig. 1c). In fact, for all 10 glycolytic enzymes, at least one isoform was significantly upregulated in SCCs relative to NC lung (Fig. S4a) and in seven of these, one isoform was significantly greater in SCCs than in AdCs.

To assess glucose utilisation, our NSCLC patients were grouped into two cohorts. In the first cohort (*n* = 15), patients were administered intravenously 10 g of uniformly ^13^C labelled glucose ([U-^13^C]-glucose) 2.8 ± 0.5 h prior to tumour resection.^13,38^ The second cohort did not receive the [U-^13^C]-glucose infusion (*n* = 16). In order to accomplish a higher degree of labelling into downstream pathways and to decouple the tissue metabolism from systemic metabolism,^31^ freshly resected tissues from both cohorts were also prepared as thin slices and incubated with [U-^13^C]-glucose for 24 h ex vivo.^13^ The ex vivo tissue slices also enabled the utilisation of more costly [U-^13^C,U-^15^N]-glutamine as a tracer.

**Fig. 2:**
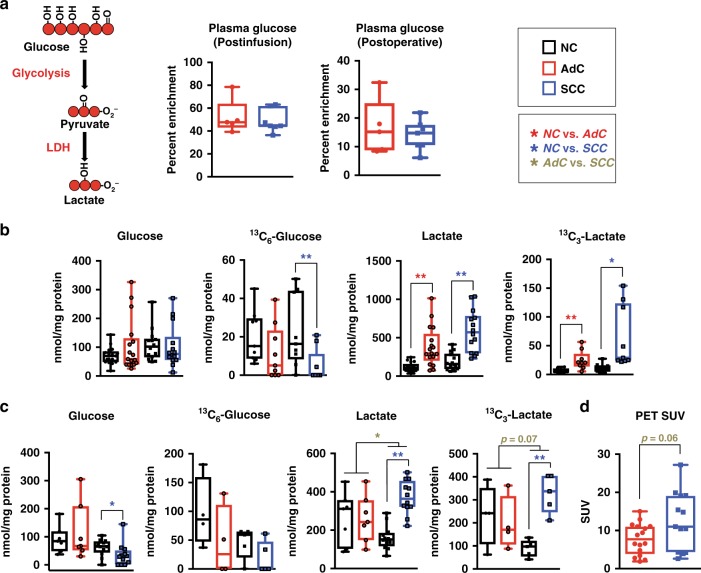
SCC tumours produce more lactate from glucose than AdC or NC. **a** Plasma samples were taken immediately after the intravenous [U-^13^C]-glucose infusion (Postinfusion) and immediately after tumour resection (Postoperative). Plasma glucose enrichment was calculated from the ^13^C satellite peaks and the unlabelled doublet for the anomeric carbon proton of α-glucose from ^1^H-NMR spectra. **b** Total and labelled glucose and lactate concentrations from primary tumours calculated from the H-C3 peak of lactate and the anomeric carbon proton of α-glucose from the proton and ^1^H{^13^C} HSQC NMR spectra, respectively. **c** Total and labelled glucose and lactate measured from tissue slices incubated with [U-^13^C]-glucose for 24 h ex vivo. For total concentration of glucose and lactate the values are combined for both groups of slices incubated with either [U-^13^C]-glucose or [U-^13^C,U-^15^N]-glutamine. **d** SUV of FDG-PET in SCC and AdC patients. See also Fig. S4 and Table S3

Plasma glucose enrichment was not different between SCC (*n* = 7) and AdC (*n* = 5) patients post-infusion or peri-operatively (Fig. 2a). Tissues from both AdCs and SCCs had reduced levels of ^13^C-glucose and greater levels of ^13^C-lactate than paired adjacent NC lung as measured by ^1^H(^13^C) heteronuclear single quantum coherence (HSQC) NMR and GC-MS (Fig. 2b and S4b), indicating that these tumours convert more glucose to lactate than NC lung. SCC tissue slices also maintained higher concentrations of total and ^13^C-enriched lactate than AdCs (Fig. 2c and S3d), which is consistent with increased levels of lactate observed in SCC tissue samples previously.^16^ Interestingly, levels of total and ^13^C-enriched lactate in NC lung tissue from SCC patients were also higher than those in NC lung resected from AdC patients (Fig. 2b). It is likely that lactate measured in the NC lung of SCC patients is at least in part derived from the tumours, as there was no increase in lactate production among NC tissues slices cultured ex vivo, while SCC tissue slices produced more lactate than their paired NC slices or AdC slices (Fig. 2c). Achieving a higher production of ^13^C-glucose-derived lactate in SCCs relative to AdCs would require both higher glucose catabolism and glucose transport. Consistently, higher standard uptake values (SUV) of ^18^F-fluorodeoxyglucose as measured by FDG-PET were observed in SCCs from our patient cohort compared with AdCs (Fig. 2d). Previous studies have demonstrated that SCCs have higher FDG-PET SUV^39,40^ as well as increased expression of glucose transporter 1 in comparison with AdCs.^41,42^

**Fig. 3:**
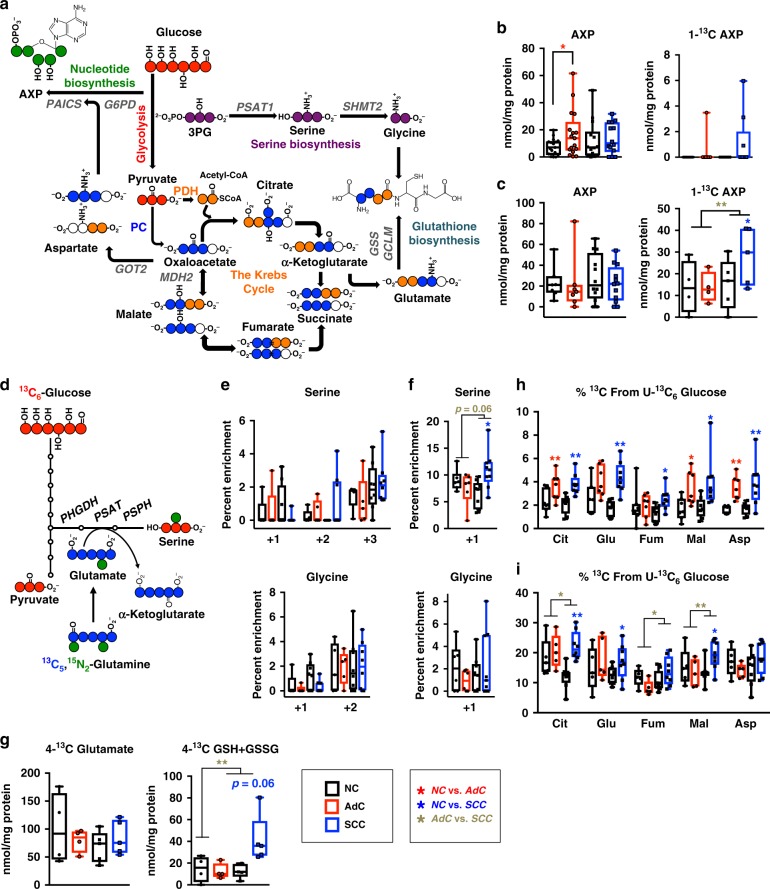
SCC tumours input more glucose-derived carbon into nucleotides, the Krebs cycle, amino acids and glutathione. **a** A diagram showing how ^13^C atoms from [U-^13^C]-glucose could be incorporated into various metabolites. **b** Levels of total and labelled adenine-containing nucleotides (AXP) calculated from the C1 position of the ribose moiety on the proton and ^1^H{^13^C} HSQC NMR spectra, respectively in resected tumours from NSCLC patients who received a [U-^13^C]-glucose bolus; **c** Levels of total and labelled AXP in tissues slices. Levels of 1-^13^C AXP measured in tissue slices incubated with [U-^13^C]-glucose ex vivo. Total levels of AXP are combined from both groups of slices incubated with either [U-^13^C]-glucose or [U-^13^C,U-^15^N]-glutamine. **d** A diagram showing how [U-^13^C,U-^15^N]-glutamine would produce ^15^N_1_-Serine. **e**, **f** Percent enrichment of serine and glycine isotopologues measured from tissues slices incubated with [U-^13^C]-glucose (**e**) or [U-^13^C,U-^15^N]-glutamine (**f**). **g** Labelled isotopomer concentrations of glutamate and glutathione (GSH) measured from the C-4 peak of glutamate on the ^1^H{^13^C} HSQC NMR spectra acquired from tissues slices incubated with [U-^13^C]-glucose. **h**, **i** Percent enrichment of citrate (Cit), glutamate (Glu), fumarate (Fum), malate (Mal) and aspartate (Asp) isotopologues measured in resected tumours from NSCLC patients receiving a [U-^13^C]-glucose bolus (**h**) and tissues slices incubated with [U-^13^C]-glucose (**i**). Data are shown as box and whisker plots where whiskers represent the min and max. *P < 0.05 and **P < 0.01. AdC and SCC tumours were compared to their paired NC counterparts using the paired *t*-test. To test for significance between AdC and SCC, the Welch *t*-test was performed on the log-ratio of the tumour to their paired NC. See also Fig. S5, S6 and Table S4

Beyond glycolysis, several enzymes in the SCC-distinguishing metabolic gene signature incorporate glucose carbons into metabolites of other pathways (Fig. 3a), and thus lactate may not be the only major destination for glucose carbon in SCC. For example, SCCs had increased expression of PPP enzymes *G6PD* and *TALDO1* and de novo nucleotide biosynthesis enzymes *UMPS, CTPS, PAICS* and *GMPS* compared to NC and AdC tissues (Fig. 1d). Higher rates of PPP and purine biosynthesis in SCCs were supported by the fact that in vivo ^13^C incorporation into the ribose subunit of adenine nucleotides (AXP) was observed in 3 out of 9 SCC, but only 1 out of 9 AdC, and was not observed in the NC counterparts (Fig. 3b). In the ex vivo tissue slices, ^13^C incorporation into AXP was observed in all but 2 NC tissues and this incorporation was increased in SCC but not in AdC slices over their paired NC tissues (Fig. 3c).

SCCs, but not AdCs, had increased concentrations of serine and glycine compared to paired NC tissues (Fig. S5a), which corresponded with the higher expression of *PSAT* and *SHMT2* in SCCs over NC and AdCs (Fig. 1c). In addition, SCCs but not AdCs overexpress 2 other enzymes in the serine biosynthetic pathway (*PHGDH* and *PSPH*, Fig. S5b). Since labelled serine and glycine were difficult to detect in vivo we turned to the tissue slice model. Serine biosynthesis from [U-^13^C]-glucose or [U-^13^C,U-^15^N]-glutamine is expected to produce serine with 3 ^13^C atoms or 1 ^15^N atom, respectively (Fig. 3d). The percentage enrichment of both was increased in SCC but not in AdC tumour slices in comparison with paired NC (Fig. 3e, f**)**, but statistical significance was only observed with [U-^13^C,U-^15^N]-glutamine as tracer (Fig. 3f).

The expression of two enzymes of de novo glutathione biosynthesis, GCLM and GSS, was higher in SCCs than in NC lungs and AdCs (Fig. 1c). Since ^13^C labelled glutathione was undetectable in vivo, it was measured in [U-^13^C]-glucose-treated tissue slices by HSQC NMR. We observed ^13^C enrichment in the C-4-glutamate moiety of glutathione (GSH+GSSG) in all but one of the paired NC slices. SCC but not AdCs tissues had significantly increased median concentration of ^13^C-4-GSH+GSSG relative to paired NC despite comparable amounts of the ^13^C glutamate precursor (Fig. 3g). Increased levels of glutathione were also previously observed in SCCs versus AdCs tissues.^16^

Glucose can label the glutamate residue of glutathione via entry of glucose-derived pyruvate into the Krebs cycle (Fig. 3a). As previously reported,^13,14^ NSCLCs oxidise more glucose through the Krebs cycle than NC lung in vivo (Fig. 3h and S6a for full isotopologue distributions). In addition, the percentage ^13^C enrichment of ^13^C-glucose-derived Krebs cycle intermediates was higher in ex vivo SCC than in ex vivo AdC tissue slices (Fig. 3i and S6b for full isotopologue distributions).

**Fig. 4:**
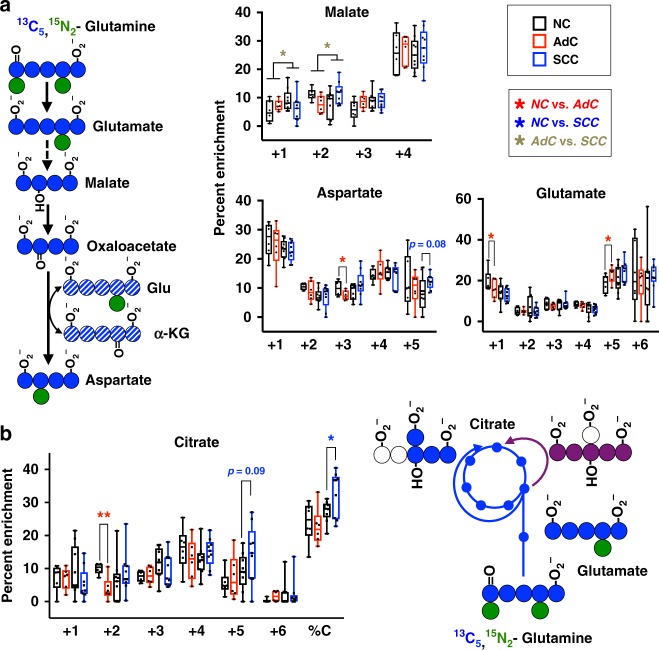
SCC tumours catabolise glutamine via transaminase reactions and reductive carboxylation. **a** A diagram shows how [U-^13^C,U-^15^N]-glutamine would produce malate m+4 and then aspartate m+4 or m+5. Percent enrichment of malate, glutamate and aspartate isotopologues in slices of tumours and NC lung incubated with [U-^13^C,U-^15^N]-glutamine for 24 h. **b** Percent enrichment of citrate Isotopologues from slices in **a**. A diagram shows [U-^13^C,U-^15^N]-glutamine would produce citrate m+4 and m+5. Data are shown as box and whisker plots where whiskers represent the min and max. *P < 0.05 and **P < 0.01. AdC and SCC tumours were compared to their paired NC counterparts using the paired or Welch *t*-test, respectively. To test for significance between AdC and SCC, the Welch *t*-test was performed on the log-ratio of the tumour to their paired NC. See also Table S5

The mitochondrial components of the malate-aspartate shuttle were also important for the separation of SCCs from NC lung and AdCs (Fig. 1c). In fact, *GOT2* was one of the most significantly overexpressed genes in every database (Fig. S1a). To track transaminase activity, slices were incubated with [U-^13^C,^15^N]-glutamine. The incorporation of ^13^C into malate and aspartate reflects glutamine entry into the Krebs cycle, which was similar for all tissue types (m+4 in Fig. 4a). However, SCCs but not AdCs tissues showed higher enrichment of aspartate containing five heavy atoms relative to paired NC lung (albeit not statistically significant), which reflects the incorporation of 4 ^13^C atoms (via oxaloacetate) plus 1 ^15^N atom from [U-^13^C,^15^N]-glutamine-derived glutamate via GOT activity^43^ (Fig. 4a).

As indicated by the percentage enrichment of ^13^C_4_-malate and -aspartate (Fig. 4a), oxidative glutamine catabolism was comparable among the tissue types. This was also evident for ^13^C_4_-citrate (Fig. 4b). Interestingly, SCCs, but not AdCs, had greater percent enrichment in ^13^C_5_-citrate than the paired NC lung, possibly the result of reductive carboxylation (Fig. 4b), which could be a consequence of the overexpression of *IDH2* in the SCC-distinguishing gene signature (Fig. S2a).

### Increased Notch activity in lung SCC correlates with the altered metabolic phenotype

**Fig. 5:**
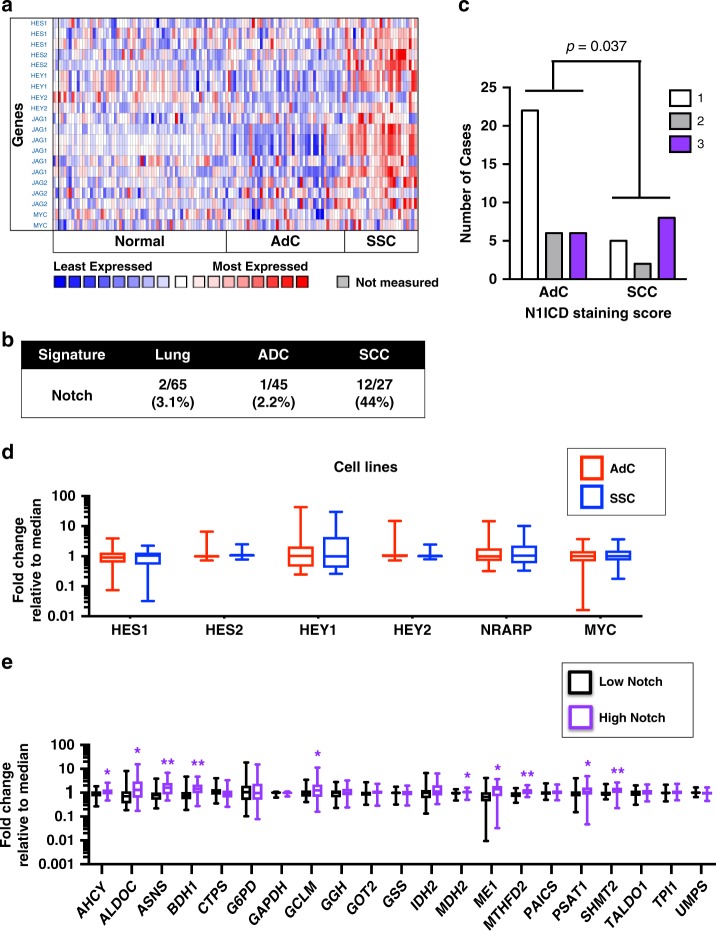
Canonical Notch signalling pathway is upregulated in human lung SCC but not AdC and correlates with the SCC-specific metabolic genes. **a** A heat map of the expression of Notch downstream targets from the Hes and Hey families and *MYC*, along with Notch ligands of the Jag family in NC, AdC and SCC tissues from the Hou database.^19^ Corroborating heat maps from additional databases can be found in Fig. S7a, c. **b** Notch pathway activation in individual samples from the Hou database^19^ based on the expression of downstream Notch targets (see Experimental Procedures for full details). **c** Nuclear activated Notch1 (N1ICD) scoring in NSCLC tissues based on immunohistochemistry reanalysed from Westhoff et al.^47^ One is low expression and 3 is high. Fisher’s Exact Test (*P* = 0.037, *χ*^2^ = 6.617 and df = 2). **d** The expression of downstream Notch targets assessed in AdC (*n* = 51) and SCC (*n* = 28) cell lines from the Broad-Novartis Cancer Cell Line Encyclopedia. Data are shown as box and whisker plots where whiskers show the min and max. *P < 0.05 and **P < 0.01. Significance was determined by two-tailed *t*-test. **e** The expression of the SCC gene signature assessed in cell lines from the Broad-Novartis Cancer Cell Line Encyclopedia with low (*n* = 39) and high (*n* = 40) expression of downstream Notch targets. Data are shown as box and whisker plots where whiskers show the min and max. *P < 0.05 and **P < 0.01. Significance was determined by two-tailed *t*-test. See also Fig. S7, Table S6, S7

Signalling pathways are known to play key roles in tumorigenesis and metabolic reprogramming. To elucidate which signalling pathways co-occur with the metabolic phenotype in SCCs, we used PANTHER Pathways and found that four pathways were enriched. The Notch pathway was the most over-represented at a 5-fold enrichment (Table S1) in SCCs compared with NC lungs and AdCs, as many of the Notch effectors and downstream targets were consistently present in the top 5% upregulated genes in SCCs (Fig. S1a). Amongst the samples in the Hou et al. database,^19^ Notch ligands *JAG1* and *JAG2* and downstream targets *HES1*, *HES2*, *HEY1*, *HEY2*^44^ and *MYC*^45^ are elevated in SCC tissues over NC lungs and AdCs (Fig. 5a). This tendency was observed in all databases (Fig. S7a, c). Notch activation was assessed in individual samples based on the expression of downstream targets (see Experimental Procedures) and was highly associated with SCC (Fig. 5b). Further, we found a high correlation between the expression of Notch pathway components and SCC-distinguishing metabolic genes (shown in black in Fig. S7b). Reanalysing data from a rigorous study that performed immunohistochemistry on 49 NSCLC patients with an antibody against the Notch1 intracellular domain (N1ICD) and quantified scoring based on its expression in the nucleus^46^ revealed that N1ICD is associated with SCC (Fig. 5c). Intriguingly, the Bhattacharjee et al.^21^ transcriptomics database had a cohort of AdCs that had increased expression of downstream Notch targets in comparison with NC lungs and the rest of AdC samples. These AdC samples also had increased expression of the 24-metabolic gene signature (highlighted in the purple box Fig. S7c) and may represent adeno-squamous tumours or adenocarcinomas with more SCC phenotype.

Elevated Notch target expression was not observed in SCC relative to AdC cell lines (Fig. 5d). Nevertheless, cells with high Notch signalling had higher expression of 10/21 SCC-distinguishing metabolic signature genes available in the Broad-Novartis Cancer Cell Line Encyclopedia (Fig. 5e).

### The relationship between Notch and the expression of metabolic genes is preserved in mouse lung tumour model

Ectopic overexpression of *N1ICD* in the mouse lung alveolar epithelium leads to the formation of adenomas with an increased Myc level and, after a long latency period, adenocarcinomas.^24^ The tumorigenicity of *N1ICD* can be augmented by the co-overexpression of *MYC*.^24^ We used this mouse model to investigate the relationship between NOTCH activation and metabolic reprogramming in NSCLC.

**Fig. 6:**
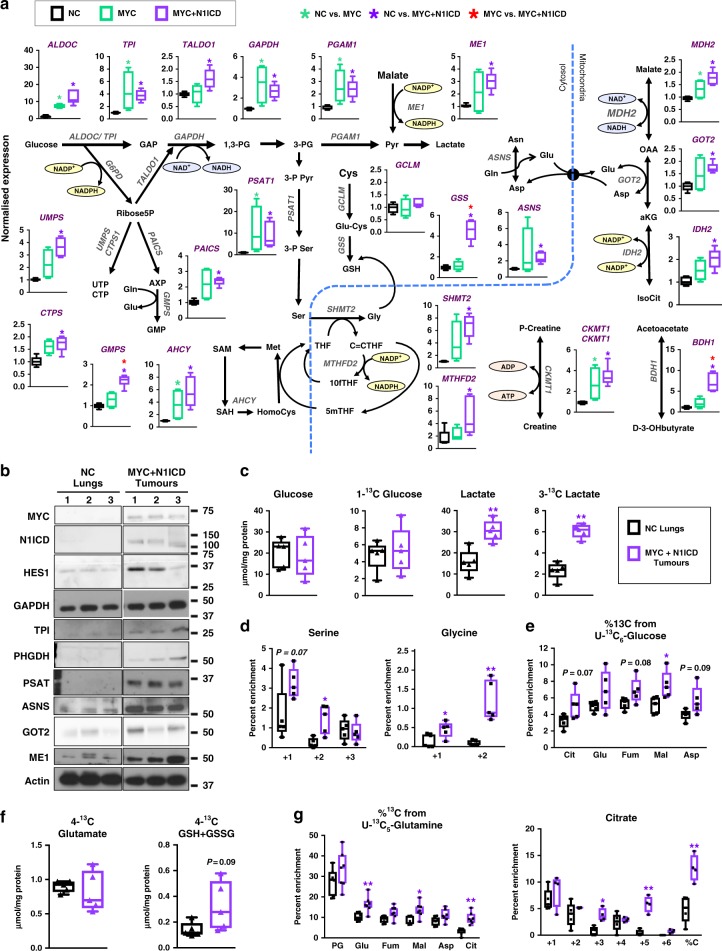
The relationship between Notch and the activity of metabolic pathways is preserved in N1ICD+MYC induced murine lung tumours. **a** Relative expression of metabolic genes measured by qPCR in *MYC+N1ICD* (purple bars, *n* = 6)*, MYC* tumours (green bars, *n* = 4) and the NC lung of mice kept off doxycycline (black bars, *n* = 6) are shown as box and whisker plots, Whiskers represent inner fences according to Tukey. **b** Protein expression of several enzymes from the SCC metabolic gene signature in *MYC+N1ICD* tumours (*n* = 3) and NC lung (*n* = 3). The Welch’s *t*-test; *P < 0.05 and **P < 0.01. **c** Percent enrichment of serum glucose in *MYC+N1ICD* (*n* = 5) tumour-bearing or control mice (*n* = 5) given a bolus injection of [U-^13^C]-glucose measured by GC-MS. The concentrations of Glc and Lac in the tumour or lung tissue were assessed by NMR as described above. **d** Percent enrichment of serine and glycine isotopologues measured in *MYC+N1ICD* tumours (*n* = 5) and control lung (*n* = 5) from mice injected with [U-^13^C]-glucose. **e** Percent enrichment of isotopologues of The Krebs cycle intermediates (citrate (Cit), glutamate (Glu), fumarate (Fum), malate (Mal) and aspartate (Asp)) in *MYC+N1ICD* tumours and control lung from mice injected with [U-^13^C]-glucose. **f** Labelled isotopomer concentrations of glutamate and GSH measured in *MYC+N1ICD* tumours and control lung from mice injected with [U-^13^C]-glucose as above. **g** Metabolite isotopologues (pyroglutamate (PG), citrate (Cit), glutamate (Glu), fumarate (Fum), malate (Mal) and aspartate (Asp)) in tissues from *MYC+N1ICD* tumour-bearing mice (*n* = 7) and NC controls (*n* = 7) injected with [U-^13^C]-glutamine. Data are shown as box and whisker plots where whiskers show the min and max. *P < 0.05 and **P < 0.01. *MYC+N1ICD* tumours were compared to control lung using Welch *t*-test. See also Figs. S8, S9, S10, S11, Tables S8, S9

We evaluated the expression of 22 of the genes in the SCC-distinguishing metabolic gene signature in *MYC+N1ICD* tumours by qPCR. We found that 21 genes were overexpressed in the *MYC+N1ICD* mouse tumours compared to NC lung from control mice (doxycycline-free diet; Fig. 6a). Among the 24-signature enzymes of human SCCs, 11 have isoforms. In most cases, one isoform is overexpressed in SCCs compared to NC lungs and AdCs (*ALDOC, BDH1, CKMT1, CTPS1, GOT2, ME1, PGAM1* and *SHMT2*, Fig. S8). In all but *SHMT*, *Myc+N1ICD* tumours upregulated the SCC-distinguishing isoform. Furthermore, both isoforms of *MDH* and *MTHFD* were elevated in SCCs compared with NC lungs and were upregulated in *MYC+N1ICD* tumours. However, isocitrate dehydrogenase did not behave the same between the human and mouse tumours. *IDH2* was upregulated in both human SCCs and AdCs, whereas all three isoforms were upregulated in *MYC+N1ICD* tumours. In summary, human SCCs specifically upregulated nine isoforms relative to NC and AdC tissues while *MYC+N1ICD* mouse tumours had the same isoform specificity in 8 out of 9 of these cases, which further supports an association between Notch activation and the SCC-distinguishing metabolism.

Since MYC is a well-known regulator of cellular metabolism,^47,48^ we next evaluated how much it might contribute to the changes in the expression of metabolic genes downstream of NOTCH. We analysed murine tumours driven by *MYC* alone.^32^ Out of 21 metabolic genes for 13 genes involved in the Krebs cycle, one carbon metabolism and nucleotide and glutathione biosynthesis, the co-expression of *MYC* and *N1ICD* led to the overexpression that was not significant in the *MYC*-only tumours and for 3 genes it enhanced the over-expression that was less pronounced in the *MYC*-only tumours (Fig. 6a). However, the expression of glycolytic and serine biosynthesis enzymes in *MYC*-only tumours was similar to *MYC+N1ICD* tumours. These results demonstrate that, although MYC can significantly contribute to the metabolic remodelling downstream of NOTCH, complete activation of the NOTCH pathway quantitatively upregulates the SCC gene signature more than MYC alone.

We next evaluated how gene expression of selected metabolic genes translates into protein expression in *MYC+N1ICD* tumours. For 7 SCC-distinguishing enzymes (ASNS, GAPDH, GOT2, IDH2, ME1, PSAT, and TPI) protein levels were increased in *MYC+N1ICD* tumours compared with the normal lungs (Fig. 6b and S9a). We also assessed the isoform pattern for GOT, ME, and IDH (Fig. S9b). The IDH1 and IDH2 protein expression patterns (Fig. S9b) were consistent with the increased expression of *IDH1* and *IDH2* mRNAs (Fig. S8). However, *GOT1*, whose mRNA expression was non-significantly increased, and *ME2*, which showed no change at the mRNA level (Fig. S8), were increased at the protein level by 3.0- and 2.9-fold, respectively (Fig. S9b).

To test how NOTCH activation affects glycolysis in NSCLC, mice bearing *MYC+N1ICD*-driven lung tumours received one bolus injection of [U-^13^C]-glucose 15 min before tumour resection. Tumours were compared to NC lungs from age-matched controls. Blood glucose enrichment was not significantly different (Fig. S10a) but tumours had higher total and ^13^C-lactate levels than NC lung (Fig. 6c and S10b). We further examined [U-^13^C]-glucose metabolism in *MYC+N1ICD* mouse tumours. Consistent with the increased expression of *PSAT* (Fig. 6a, b), *MYC+N1ICD* tumours had greater ^13^C incorporation into ^13^C_1_- and ^13^C_2_-serine (Fig. 6d and S10c**)**, presumably produced by the Ser/Gly exchange reaction catalysed by SHMT (see Fig. S5c for full description) from ^13^C_2_-glycine than NC lung (Fig. 6d). *MYC+N1ICD* tumours also had increased glucose incorporation into the Krebs cycle, although the increase did not reach significance (Fig. 6e and S10d for full isotopologue distributions). Like SCCs, *MYC+N1ICD* tumours had elevated levels of ^13^C incorporation into the 4-^13^C position of the glutamate residue of GSH+GSSG, despite having similar levels of the labelled glutamate precursor (Fig. 6f), which was consistent with the increased RNA levels of *GSS* (Fig. 6a).

When the *MYC+N1ICD* mice were injected with [U-^13^C]-glutamine, blood glutamine enrichment was not significantly different (Fig. S11a) but Krebs cycle intermediates in *MYC+N1ICD* tumours showed increased percentage of ^13^C enrichment, particularly for ^13^C_5_-citrate (Fig. 6g), reminiscent of the SCC tissue slices (Fig. 4b). *MYC+N1ICD* tumours also displayed a greater catabolism of ^13^C-glutamine into ^13^C_4_-fumarate and -malate (oxidative route, Fig. 6g) and ^13^C_3_- fumarate and –malate. The latter likely derived from ATP citrate lyase-catalysed cleavage of ^13^C_5_-citrate produced via reductive carboxylation and/or malic enzyme activity on ^13^C_4_-malate to produce ^13^C_3_-pyruvate, which can be carboxylated to ^13^C_3_-oxaloacetate by pyruvate carboxylase (Fig. S11b). This was reproduced in a second cohort of animals (Fig. S11c).

Together, the data support a relationship between NOTCH activation and the metabolic reprogramming observed preferentially in SCC. They also suggest that MYC can be one of the major mediators of the effect of NOTCH on the expression of metabolic genes. However, other signalling pathways likely cooperate or converge with Notch to meet the evolving metabolic requirements of tumour cells. Indeed, Notch1 expression and activity of Notch pathway was shown to be downstream of Nrf2,^49,50^ which is mutated in higher percentage of SCCs than ADCs^51^ and was shown to regulate the expression of metabolic enzymes.^52^

Together, our data point to a complex interaction between the tumour microenvironment, cell signalling and metabolism, emphasising the need to study lung cancer metabolism in vivo.

## Discussion

The integration of transcriptomic and metabolomic data revealed a metabolic reprogramming signature associated with SCC. We show that SCCs had a more active catabolism of glucose carbon into a variety of pathways including glycolysis, the Krebs cycle, and nucleotide, amino acid and glutathione biosynthesis, which was supported by the higher expression of relevant enzymes. Our results demonstrate that the metabolic differences between subtypes are the result of the whole programme, which can be driven by Notch among other signalling pathways.

The activity of many metabolic pathways that distinguish SCC from AdC tumours, including glycolysis, PPP, the Krebs cycle and nucleotide biosynthesis, are required to support higher proliferation, as previously reported for SCC compared with AdC tumours.^39,53,54^ It is also consistent with both NOTCH1 and MYC being master regulators of cell proliferation.^47,55^ At the same time other differences, like higher reductive carboxylation of glutamine, may reflect differences in other cell autonomous functions or the tumour microenvironment.

For instance, these pathways not only provide the carbon for biosynthetic precursors but can also supply glutathione and the reducing equivalent NADPH for ROS detoxification (Fig. S12) and fatty acid synthesis. Moreover, both cytoplasmic and mitochondrial isoforms of *GOT* and *MDH* were elevated in the SCCs over NC lung and AdCs, which suggests that these tumours have a higher activity of the malate-aspartate shuttle, possibly to maintain NAD^+^ homeostasis. The simultaneous increase in glycolytic and the Krebs cycle activity in SCC may require enhanced malate-aspartate shuttle to deliver NADH produced by glycolysis into the mitochondria for oxidation and to regenerate cytoplasmic NAD^+^ to support further glycolytic activity.^56^ Finally, we observed that glutamine contributed a substantial amount of carbon to the Krebs cycle in both normal lung and tumours; however, SCCs may catabolise glutamine reductively which should be investigated further with the appropriate tracer to evaluate its quantitative importance.

Our results also demonstrated that the Notch signalling pathway was highly enriched in SCCs and Notch activity correlated strongly with the observed metabolic phenotypes. However, the role of Notch in lung cancer is controversial. The Cancer Genome Atlas found that 8% of SCCs carry mutations in *NOTCH1*, half of which are predicted to be truncating suggesting a tumour suppressive function of Notch1.^20^ This prediction is inconsistent with an earlier study that functionally characterised the 4 *NOTCH1* mutations observed in their cohort NSCLC patients and concluded Notch mutations were gain-of-function.^46^ Notch1 expression and activity in NSCLC has been extensively studied via immunohistochemical staining of human tumours and in vitro studies with established cell lines, with seemingly contradictory results (recently reviewed^57^). These studies typically assessed Notch staining both in the membrane/cytoplasm and nucleus and therefore can only make limited conclusions with regards to the Notch transcriptional activity. This is in contrast to our study, which examined mRNA expression of downstream Notch targets and, therefore, should be more indicative of the Notch activity than the Notch1 protein expression levels alone.

In our study, the oncogenic and metabolic function of Notch1 in human SCC was supported by the murine model (*MYC+N1ICD*) we used. While *N1ICD+MYC* tumours resembled the metabolic phenotype observed in human SCCs, comparing the expression of metabolic genes in *N1ICD+MYC* lung tumours and lung tumours induced by *MYC* alone showed that the expression of some signature enzymes was similar between the two types of tumours. This suggests that Notch is unlikely to directly regulate the expression of all 24-metabolic signature genes and that *MYC* can be one of the mediators of Notch’s effect on metabolism in our models.

Lastly, differential regulation of metabolic pathways in NSCLC subtypes may suggest differential metabolic vulnerabilities and potential therapeutic targets. The metabolic gene signature might also explain the differential efficacy of gemcitabine over pemetrexed in SCC compared to AdC when paired with cisplatin^58^ and the preferred choice of combined gemcitabine and a platinum-based drug for first-line treatment of SCC patients.^59^ First, the chemotherapeutic agent gemcitabine is a potent competitive inhibitor of the SCC-signature enzyme CTPS1 and this activity may potentiate gemcitabine toxicity by amination of the inactive uracil metabolite 2′-deoxy-2′, 2′-difluorouridine triphosphate.^60^ Secondly, the lower sensitivity of SCCs to pemetrexed, an anti-folate drug, has been suggested to be associated with the higher activity of another SCC-signature enzyme, GGH.^61^ GGH hydrolyses the active polyglutamated version of pemetrexed.

As our signature may explain the differential efficacies of current standard of care chemotherapies, it may also reveal novel vulnerabilities. Higher expression of IDH2 together with higher reductive carboxylation of glutamine in SCCs in comparison with AdCs may suggest higher levels of ROS and consequent necessity for antioxidant protection.^62^ This is also consistent with increased levels of glutathione and GSS in SCCs but not in AdCs. Therefore, targeting both IDH2 and glutathione biosynthesis may prove to be efficient specifically in SCCs. Furthermore, the sensitivity of tumours to serine/glycine-deficient diet has recently been suggested to depend on their ability to synthesise serine and glycine from glucose.^63^ Increase in serine biosynthesis in SCCs but not in AdCs suggests that AdCs maybe more sensitive to a serine/glycine deficient diet but at the same time SCCs may be addicted to serine biosynthesis.

Importantly, both the metabolic signature-based separation of SCC and AdC histotypes and association of increased Notch pathway activity in SCC tumours are lost in established cancer cell lines. Although recent data from Goodwin and co-authors demonstrate that AdC and SCC cell lines differ in their glucose catabolism,^42^ our results suggest that many metabolic aspects are lost in in vitro versus in vivo systems. This can be due to cells adapting to growing on plastic being exposed to non-physiological concentrations of oxygen and nutrients or loss of intratumoral cellular interactions or both. Our results strongly argue that in vivo analyses of metabolism are required to understand the relationship between histotypes, metabolism and oncogenic drivers.

## Supplementary information

Table S1

Table S2

Table S3

Table S4

Table S5

Table S6

Table S7

Table S8

Table S9

Table S10

Table S11

Table S12

Table S13

Table S14

Table S15

Table S16

Table S17

Table S18

Table S19

Supplemental Information

## Data Availability

The data sets generated during and/or analysed during the current study are available from the corresponding author on reasonable request.
