# Supplementary material for: Metabolic reprogramming and Notch activity distinguish between non-small cell lung cancer subtypes
Source: Br J Cancer. 2019 May 22;121(1):51–64. doi: 10.1038/s41416-019-0464-z (PMC6738087; doi:10.1038/s41416-019-0464-z)
Supplement: Supplementary file 1 — Table S1 [file 41416_2019_464_MOESM1_ESM.docx]

**Table S1: Significantly enriched pathways in SCC tumors compared to NC lung and non-SCC lung cancer histotypes.**

Oncomine^TM^ was used to extract the top 5% upregulated genes in SCC from four databases ^18-21^. Genes that overlapped in at least three of the four databases were analyzed by PANTHER Gene Ontology based on gene ontology biological processes or PANTHER pathways. This table lists all the significantly enriched pathways.
