# Supplementary material for: Metabolic reprogramming and Notch activity distinguish between non-small cell lung cancer subtypes
Source: Br J Cancer. 2019 May 22;121(1):51–64. doi: 10.1038/s41416-019-0464-z (PMC6738087; doi:10.1038/s41416-019-0464-z)
Supplement: Supplementary file 20 — Supplemental Information [file 41416_2019_464_MOESM20_ESM.pdf]

Figure S1: Metabolic enzymes are a significant class of genes upregulated in lung squamous cell carcinoma (SCC) across databases. Related to Figure 1.

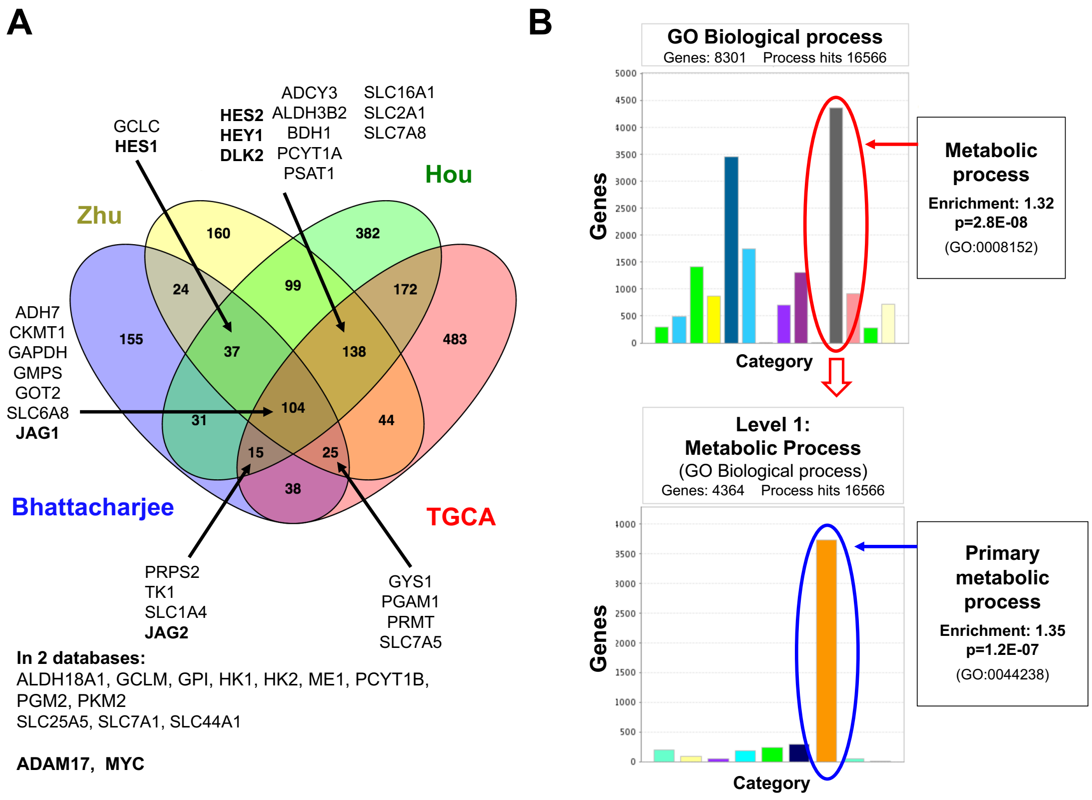

A. Oncomine™ was used to extract the top 5% upregulated genes in SCC from several databases [18-21]. The Venn diagram shows the genes that recur in multiple databases. SCC-specific genes that participate in central carbon metabolism are listed. Canonical Notch signaling pathway effectors also reoccur (highlighted in bold). **B.** Genes that overlapped in at least three of the four databases were analyzed by Panther Pathway Analysis. Metabolic processes (grey bar circled in red) constituted the bulk of overlapping genes. Metabolic processes, and specifically primary metabolic processes (orange bar circled in blue), were the third and fourth most significantly enriched pathways, respectively.

Figure S2: Primary squamous cell carcinoma (SCC) has a distinct metabolic profile from non-cancerous (NC) lung tissue and adenocarcinoma (AdC) that is not maintained in cell culture. Related to Figure 1.

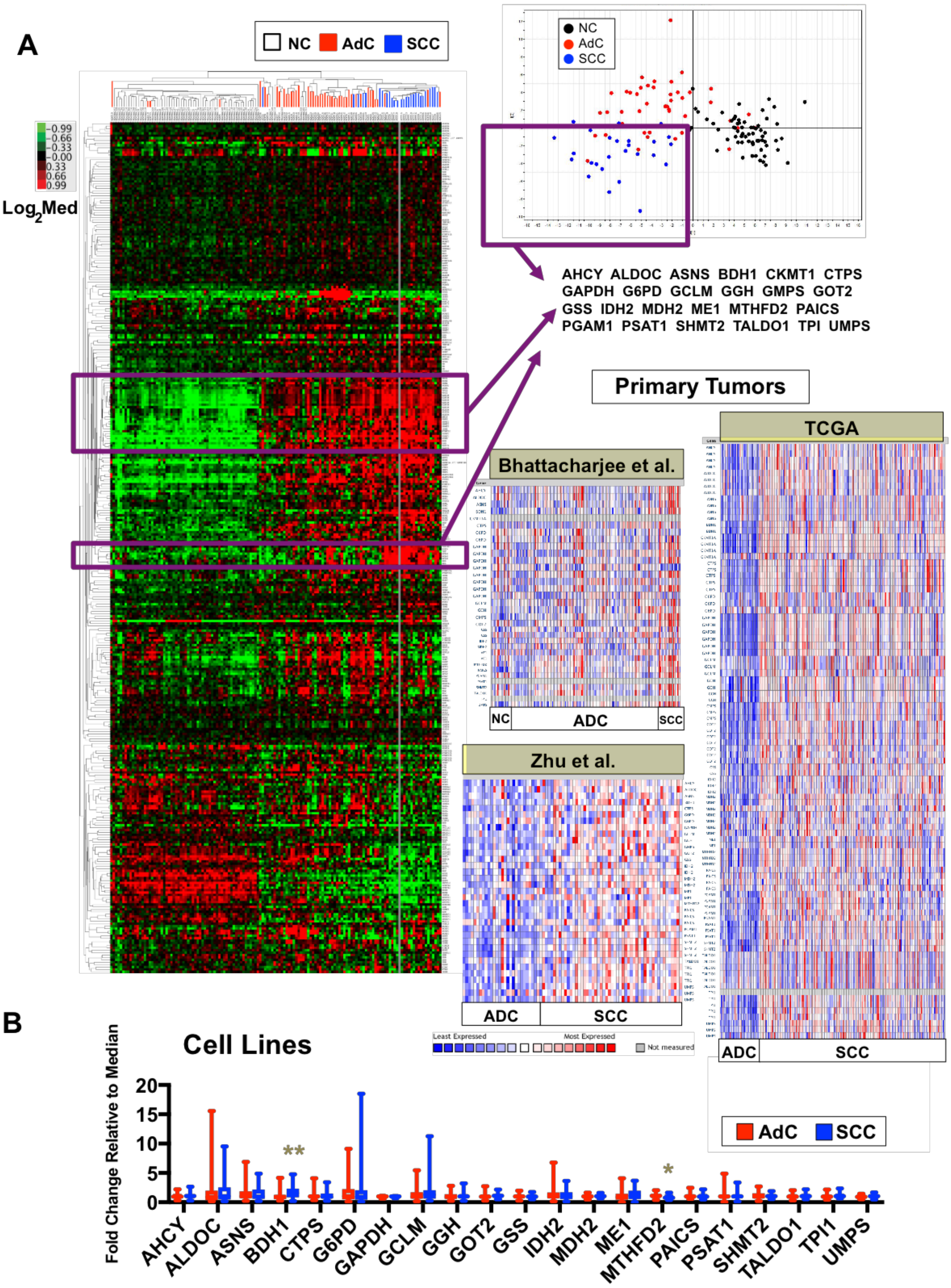

A: Hierarchical clustering of the expression of enzymes that participate in central carbon metabolism in AdC, SCC and NC lung tissues from the Hou et al. database [19]. Two clusters contain enzymes that are upregulated in SCC relative to NC or AdC. In PCA, SCC separates from NC lung tissues and AdC tumors in the first and third components, respectively. 24 enzymes are found both in the SCC-defining clusters and are responsible for the separation of SCC in both PC1 and PC3. The expression of these 24 genes were analyzed in three other lung cancer databases, where the information was available. **B:** The expression of the SCC gene signature assessed in AdC

(n=51) and SCC (n=28) cell lines from the **Broad-Novartis Cancer Cell Line Encyclopedia**. Data are shown as box and whisker plots where whiskers show the min and max. * - P <0.05 and ** - P <0.01. Significance was determined by two-tailed t-test. See **Table S10**.

Figure S3. Metabolic gene signature correlates with the NCSL tumor stage and is predictive of a worse overall survival. Related to Figure 1.

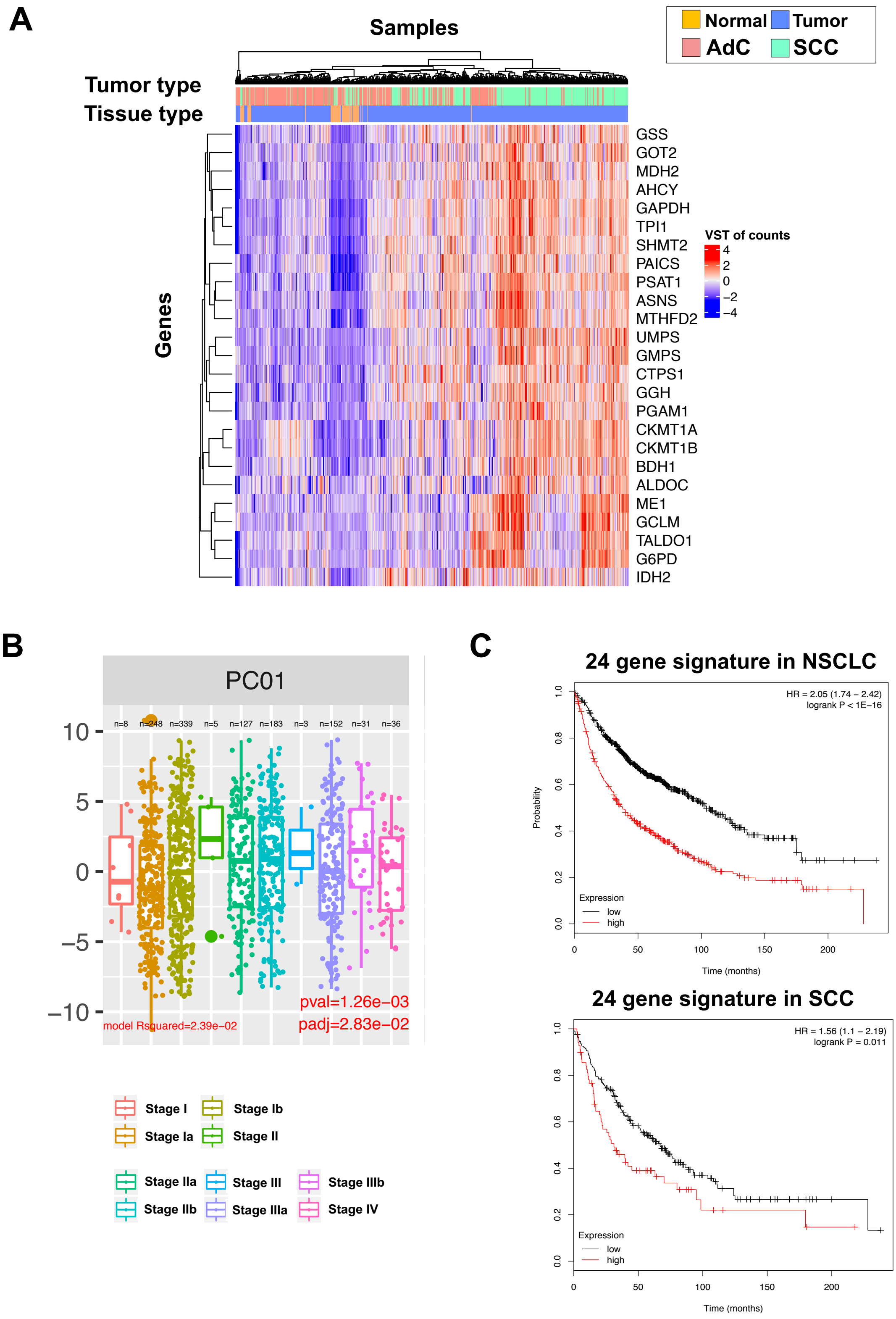

A: Heatmap of the 25 genes signature in lung cancer (lung adenocarcinoma and lung squamous cell carcinoma). In contrast to microarray data, the expression of CKTM1A and CKMT1B genes was quantified separately. Heatmap showing the Z-scores of the VST counts of the gene signature for each patient sample.

The rows and columns were clustered using the euclidean distance with complete agglomeration method. **B:** The principal component analysis was performed on the VST counts of the gene signature then an ANOVA was performed on the PCs values between the tumor stage groups. The first principal component is presented. The bigger dots indicate outliers. For each group, the number of samples is annotated on the top. The p-values and adjusted p-values of the ANOVA are annotated at the bottom right. The R squared of the model is annotated at the bottom left. A red color font indicates an adjusted p-value lesser than 0.05. **C:** Association between high expression of the 24 SCC gene signature and survival among NSCLC and SCC patients assessed by KM plotter [25].

Figure S4. SCC tumors have higher expression of glycolytic enzymes relative to AdCs and NC lungs and glucose uptake appears to be uncoupled from lactate production. Related to Figure 2.

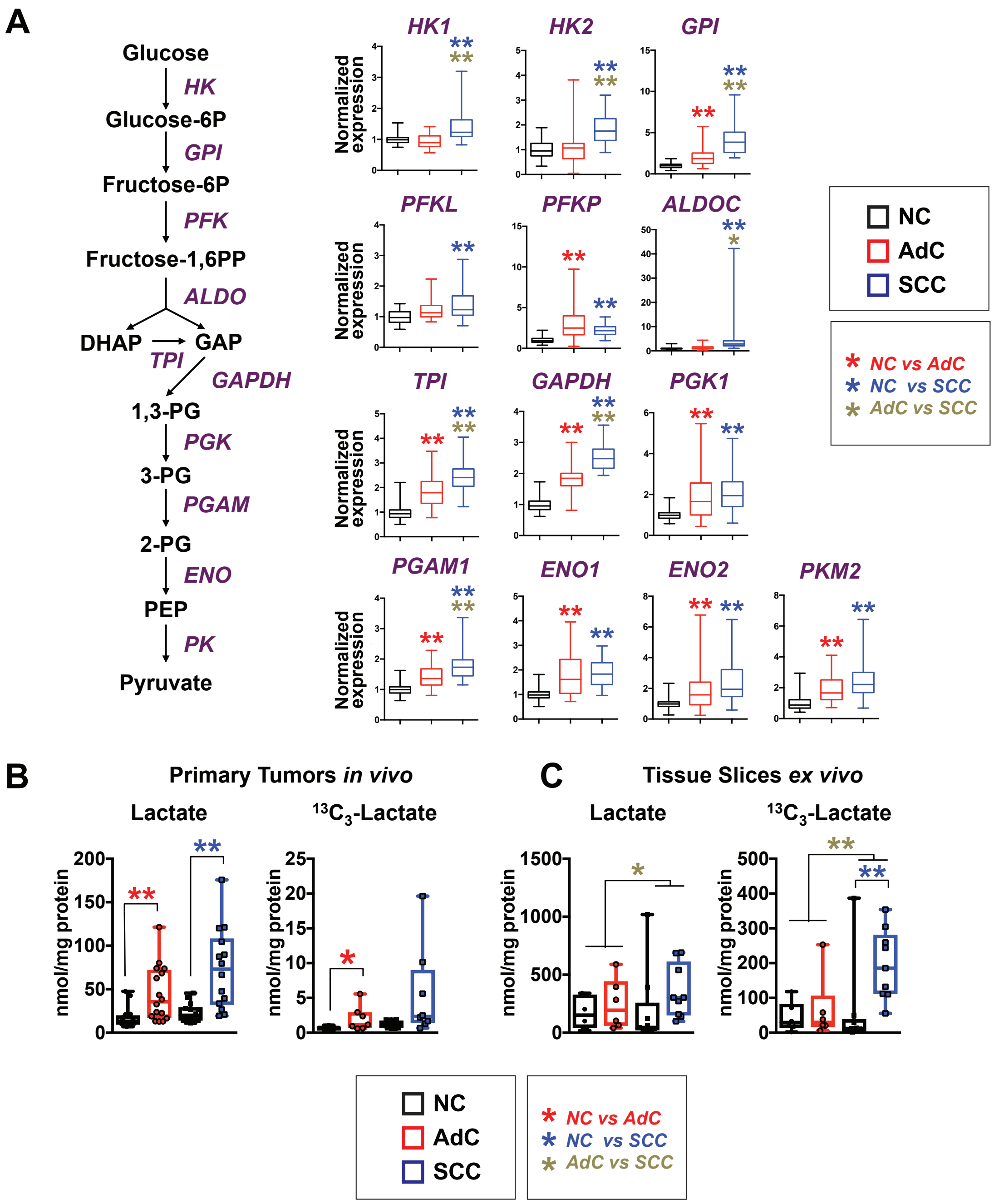

A: Relative expressions of glycolytic genes shown as box and whisker plots for AdCs (n=45) and SCCs (n=27), normalized to the median expression in NC lungs (n=65). Whiskers represent min to max. One-way ANOVA with Tukey correction for multiple comparisons between tissue groups and Bonferroni correction for multiple comparisons of different genes. * - P <0.05 and ** - P <0.01. **B:** Concentrations of total and $^{13}C_3$-lactate measured by GC-MS in primary tumors resected from patients receiving a bolus injection of [U-^{13}C]-glucose. For total concentration of glucose and lactate the values are combined for both groups of slices incubated with either [U-^{13}C]-glucose or [U-^{13}C,U-^{15}N]-glutamine. **C:** Concentrations of total and $^{13}C_3$-lactate measured by GC-MS in tissue slices incubated with [U-^{13}C]-glucose for 24h *ex vivo***.** Data are shown as box and whisker plots where whiskers show the min and max. * - P <0.05 and ** - P <0.01. AdC and SCC tumors were compared to their paired NC counterparts using the paired t-test. To test for significance between AdC and SCC, the Welch t-test was performed on the log-ratio of the tumor to their paired NC. See **Table S11** and **S12**.

Figure S5. SCC tumors upregulate the expression of serine/glycine biosynthetic enzymes. Related to Figure 3.

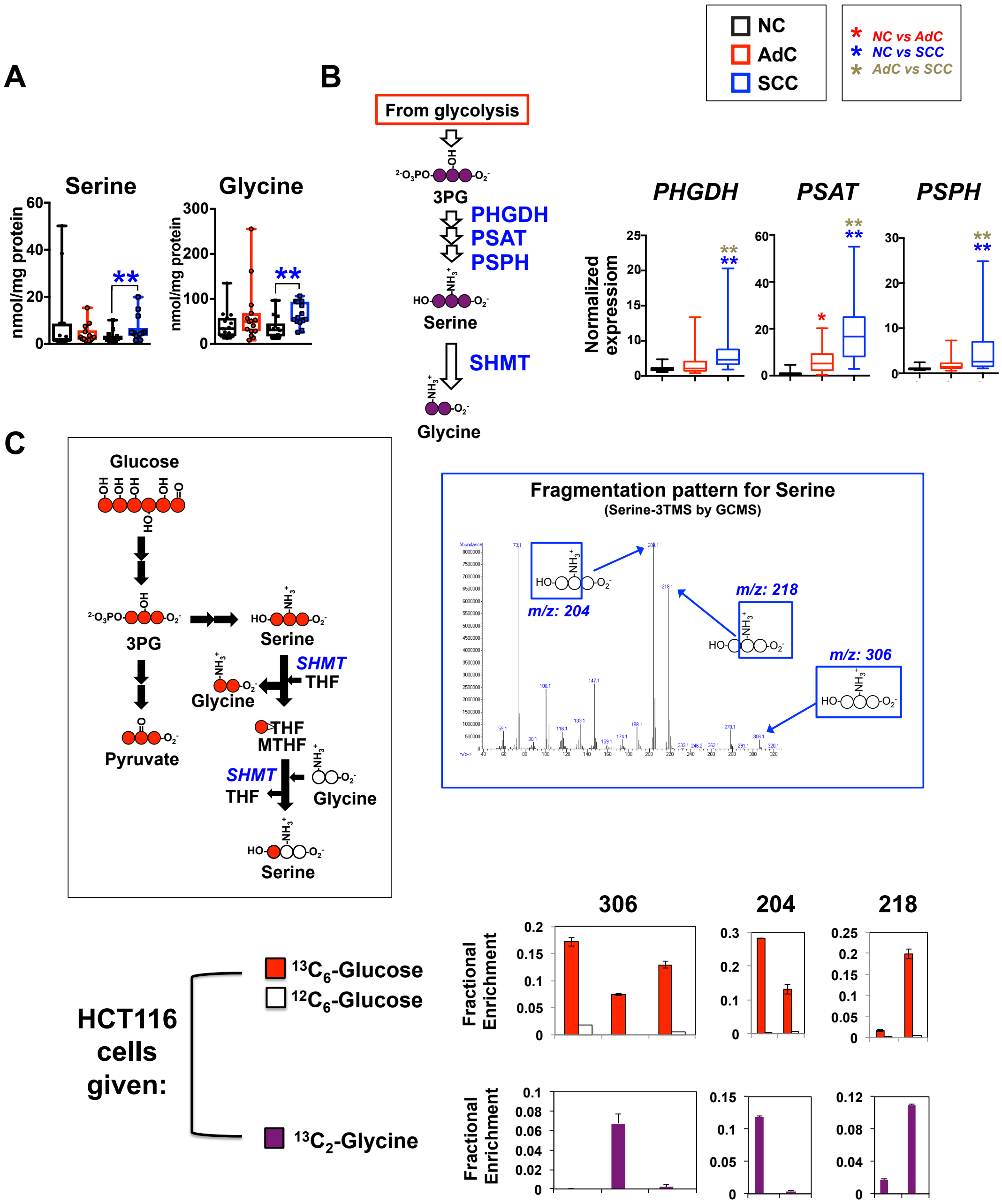

A: Total serine and glycine levels quantified by GC-MS in primary NSCLC tumors. **B:** Relative expressions of serine biosynthesis enzymes shown as box and whisker plots for AdCs (n=45) and SCCs (n=27), normalized to the median expression in NC lungs (n=65). Whiskers represent min to max. One-way ANOVA with Tukey correction for multiple comparisons between tissue groups and Bonferroni correction for multiple comparisons of different genes. * - P <0.05 and ** - P <0.01. **C:** The GC-MS fragmentation pattern for Ser. Highlighted are fragments that contain all 3 carbons (m/*z* 306), C1 and 2 carbons (m/*z* 218), and C2 and 3 carbons (m/*z* 204). Enrichment patterns are shown for each fragment after HCT116 cells were given no labeled tracer, [U-^{13}C]-glucose or [U-^{13}C]-glycine. A diagram shows how reverse flux through SHMT on glucose-derived glycine or 5-THF can label serine at carbons 1 and 2 or at carbon 3, respectively. See **Table S13** and **S14**.

Figure S6. Incorporation of [U-^{13}C]-glucose into TCA cycle intermediates in human tumors. Related to Figure 3.

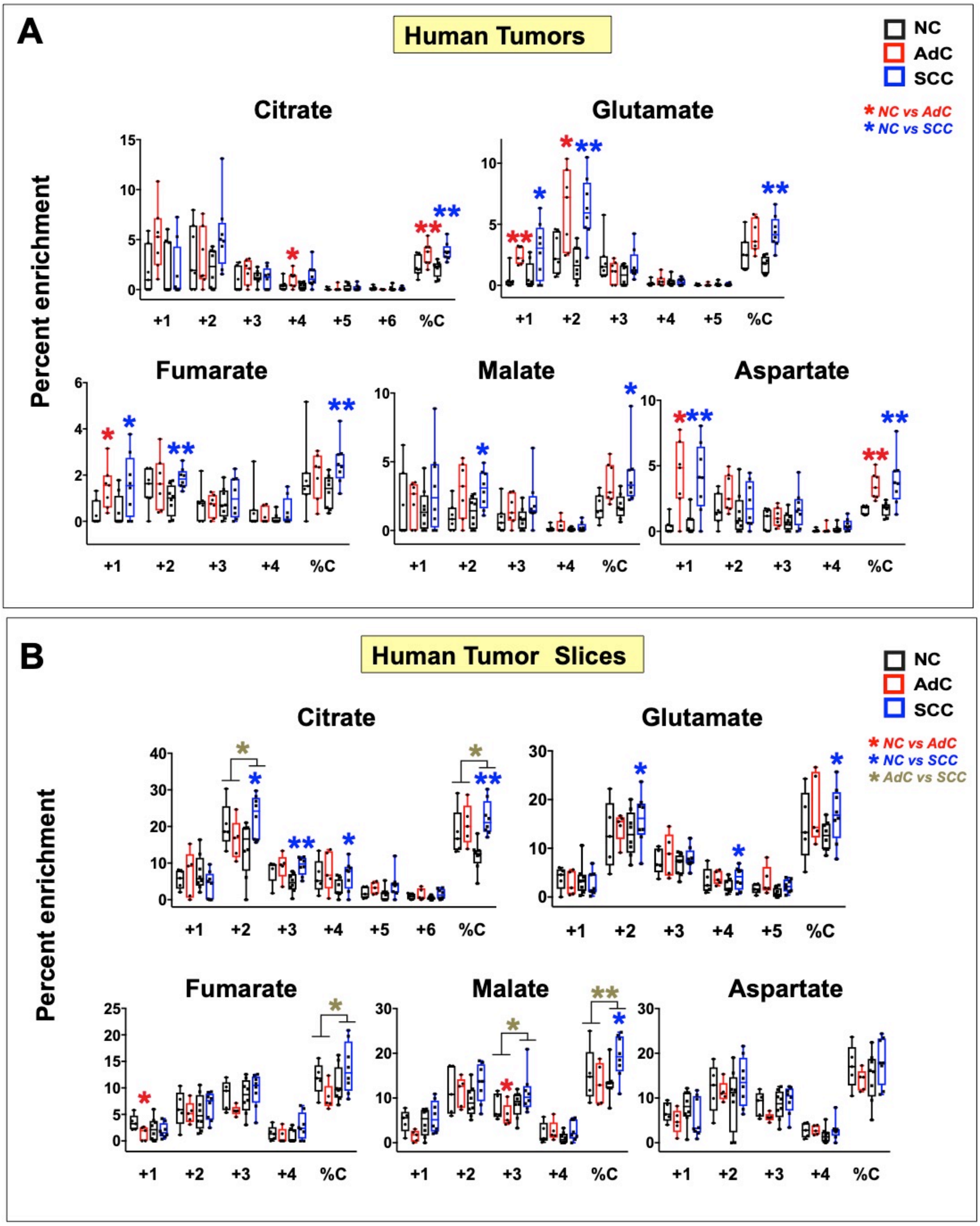

Citrate, glutamate, fumarate, malate, and aspartate measured in tumors from patients who received a [U-^{13}C]-glucose bolus **A**) and tissues slices incubated with [U-^{13}C]-glucose *ex vivo* **B**). Data are shown as box and whisker plots where whiskers represent the min and max. * - P <0.05 and ** - P <0.01. AdC and SCC tumors were compared to their paired NC counterparts using the paired t-test. To test for significance between AdC and SCC, the Welch t-test was performed on the log-ratio of the tumor to their paired NC. See **Table S15**.

Figure S7: Canonical Notch signaling pathway is overexpressed in primary human lung SCCs relative to AdCs but not in established cell lines. Related to Figure 5.

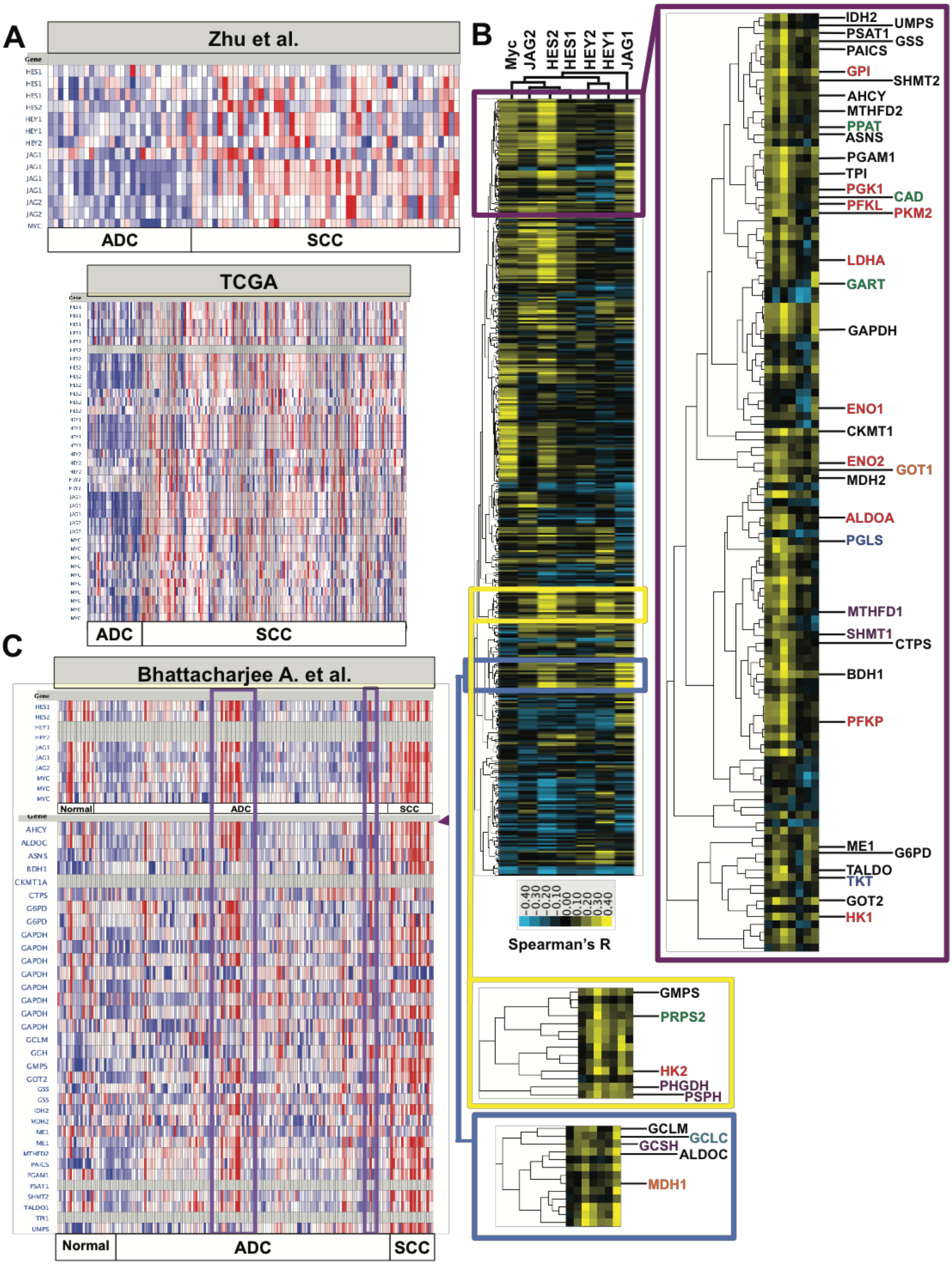

A: Heat maps from two lung cancer databases show the expression of Notch downstream targets from the Hes and Hey families and *MYC*, along with Notch ligands of the Jag family. **B:** The co-expression of Notch targets and ligands and metabolic enzymes in the Hou et al. database [19] assessed with Spearman's Rank Correlation and

visualized by clustering based on Spearman's R values. The cluster of genes highlighted in purple shows genes that correlated with Notch pathway genes including *MYC* and the clusters highlighted in yellow and blue are genes that correlated with Notch pathway genes excluding *MYC*. All of the genes from the 24 SCC metabolic signature are within these clusters (shown in black), along with several other enzymes in the pathways the signature genes participate such as glycolysis (shown in red), nucleotide biosynthesis (shown in green), pentose phosphate pathway (shown in blue), seine-glycine biosynthesis and one carbon metabolism (shown in purple), and the malate-aspartate shuttle (shown in orange). **C:** Bhattacharjee at al. database [21] contains a cohort of AdC tumors with high expression of Notch targets and ligands. This heat map is alignment with the heat map showing expression of the SCC metabolic gene signature (lower map, also shown in **Figure S2**).

Figure S8: Gene expression patterns of isoforms in the metabolic reprograming of human SCC tumors match that of mouse tumors driven by *MYC+N1ICD*. Related to Figure 6.

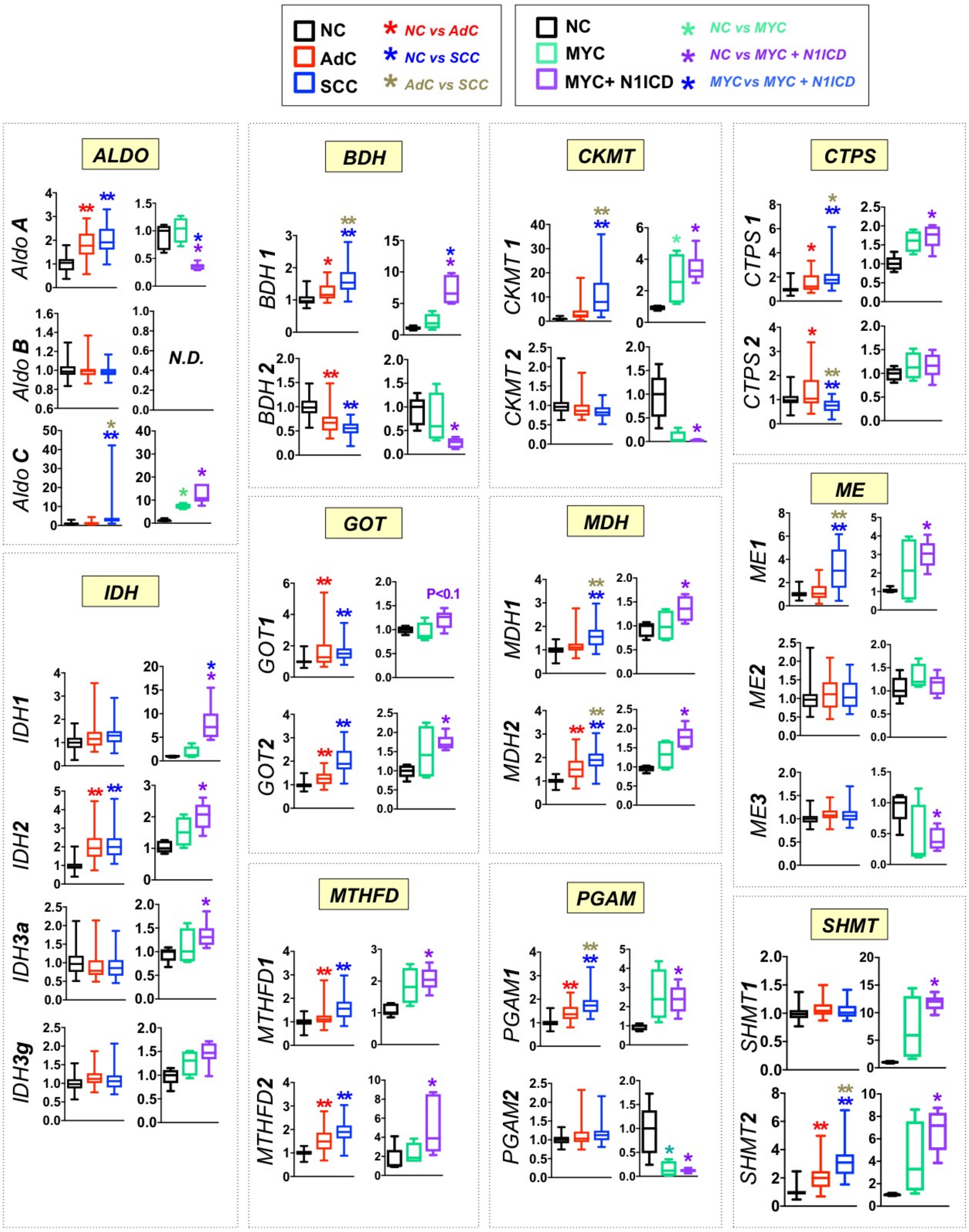

11 of the enzymes in the SCC metabolic gene signature have isoforms. Their expression is compared for human (the Hou database (Hou et al., 2010)) and mouse (measured by qPCR) genes. For instance, there are three isoforms of aldolase (*ALDOA*, *B*, and *C*). The ubiquitously expressed glycolytic isoform *ALDOA* is upregulated in both AdC and SCC tumors, whereas the brain-specific isoform *ALDOC* is overexpressed only in human SCC lung tumors (blue bars, n=27) relative to NC lungs (black bar, n=65) or AdC tumors (red bars, n=45). Likewise, it is specifically *ALDOC* that is upregulated in the *MYC+N1ICD*-driven tumors (purple bars, n=6) compared to control lung (black bars, n=6). The specificity can be seen in the expression of many of the isoform sets. Significance was determined using one-way ANOVA with Tukey correction for multiple comparisons or the Welch's t-test for the human and mouse data, respectively. * - P <0.05 and ** - P <0.01. See **Table S16**.

Figure S9. Protein expression of enzymes in *MYC+N1ICD* tumors partially matches gene expression. Related to Figure 6.

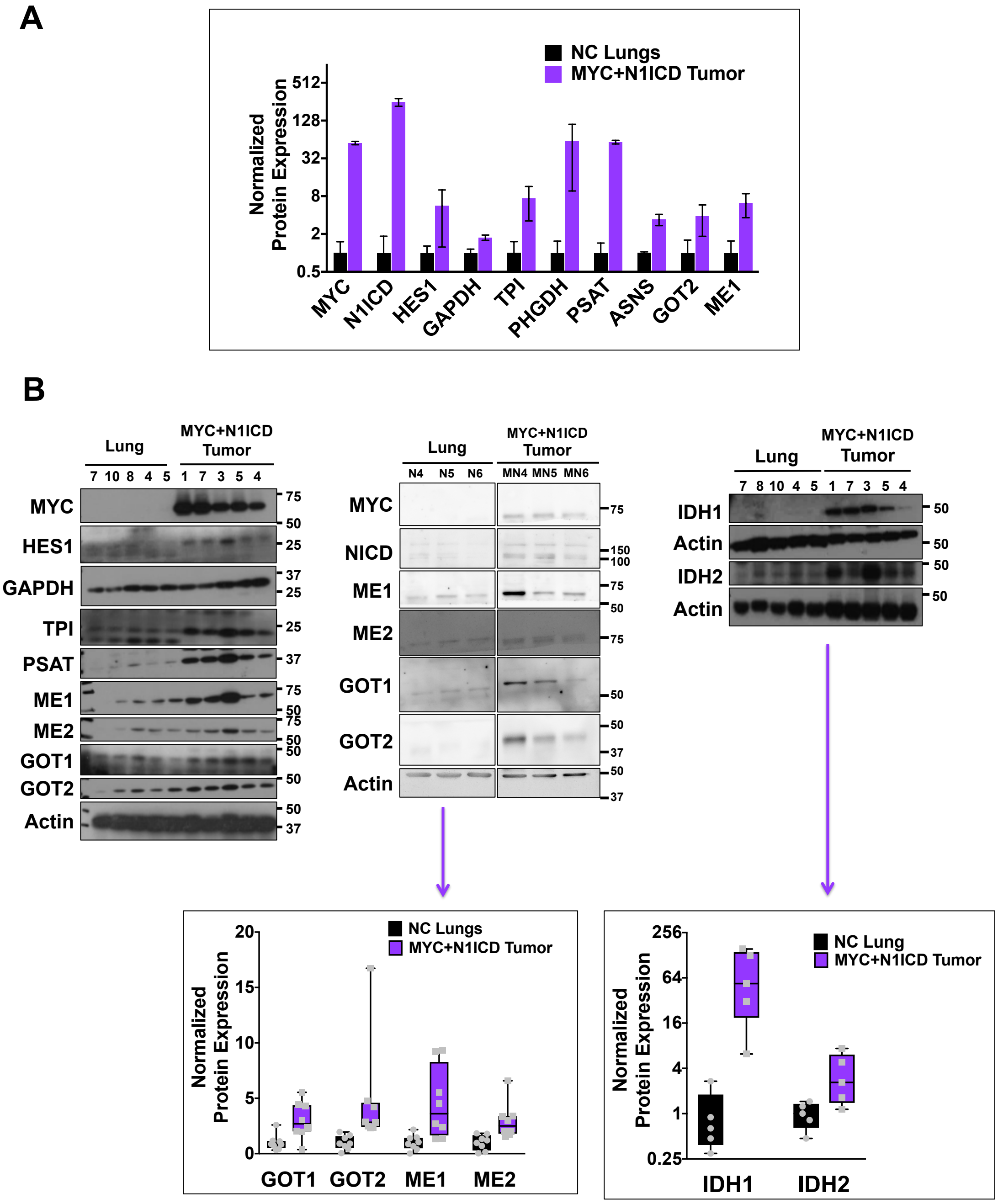

A) Densitometry of protein expression of enzymes from Figure 6B was measured by ImageJ and normalized to Actin. **B**) Protein expression of several enzyme isoforms from the SCC metabolic gene signature in *MYC+N1ICD* tumors and the quantification of the protein expression from two presented blots combined, *MYC+N1ICD*-driven tumors (n=8) and NC lungs (n=8). See **Table S17**.

Figure S10. Incorporation of [U-^{13}C]-glucose into lactate, serine and TCA cycle intermediates in *MYC+N1ICD* mouse tumors. Related to Figure 6.

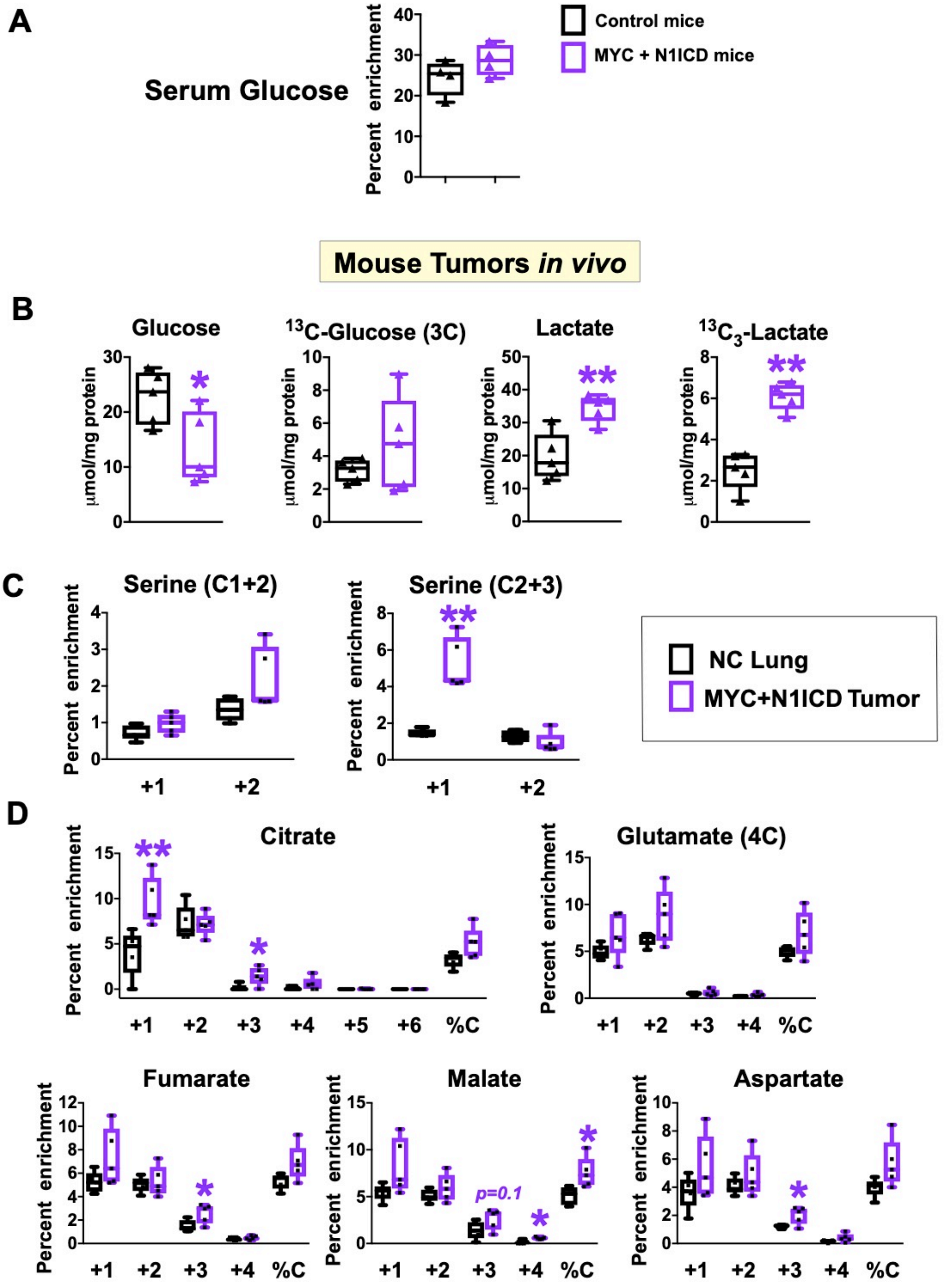

Serum glucose levels **A)**, concentrations of total and $^{13}C_3$-lactate or $^{13}C_3$-glucose **B)**, serine isotopologues **C**), and TCA intermediates (citrate, glutamate, fumarate, malate, and aspartate) **D)** measured by GC-MS in *MYC+N1ICD* tumors (n=5) or control lungs (n=5) from mice given a bolus injection of [U-^{13}C]-glucose. A fragment of glutamate containing only 4 of the original glutamate carbons was chosen for quantification because it was more abundant and had less interference from other peaks. Data are shown as box and whisker plots where whiskers show the min and max. * - P <0.05 and ** - P <0.01. *MYC+N1ICD* tumors were compared to control lung using the Welch t-test. See **Table S18**.

Figure S11. Incorporation of [U-^{13}C]-glutamine into TCA cycle intermediates in *MYC+N1ICD* mouse tumors. Related to Figure 6.

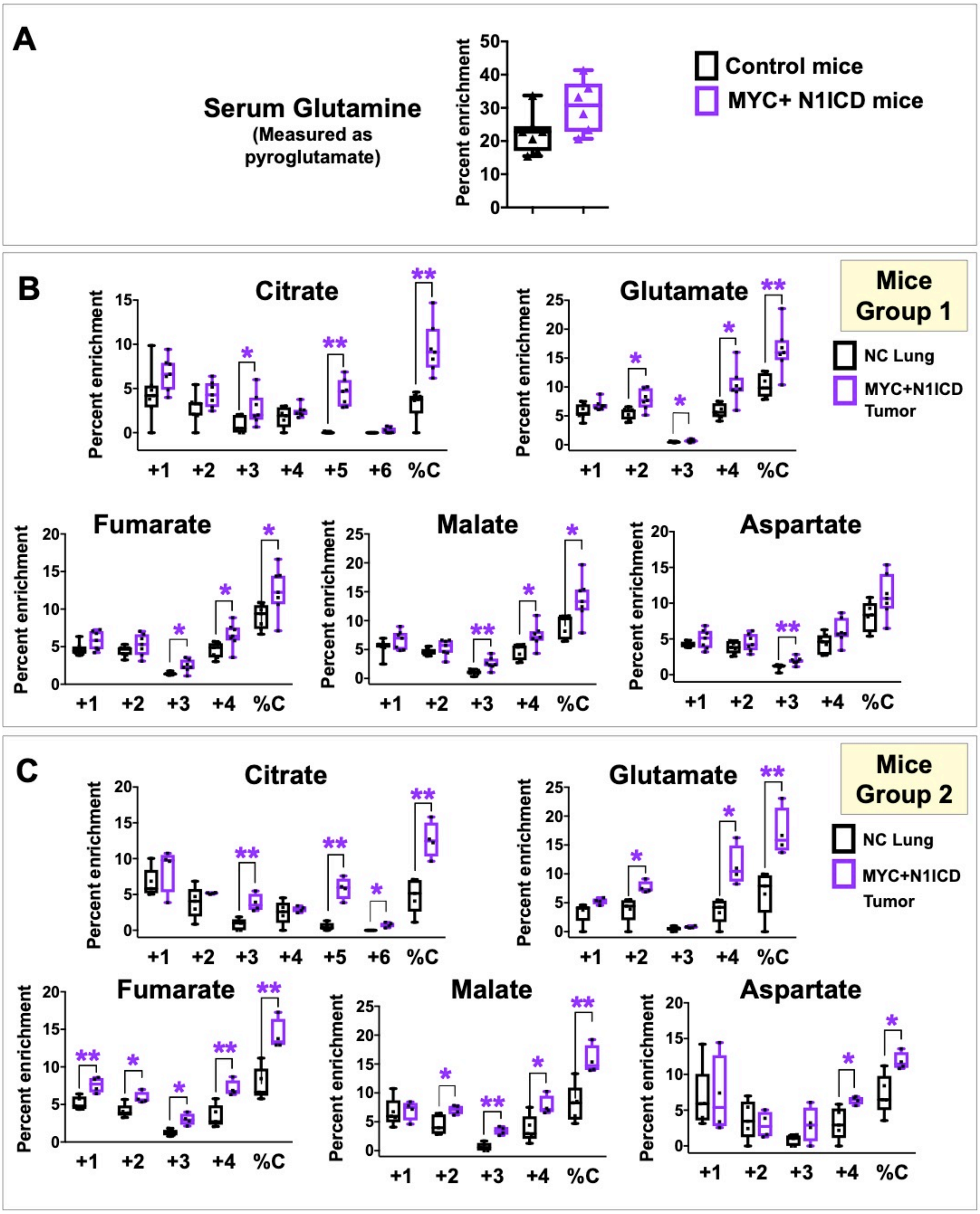

Serum glutamine levels **A)** and concentrations of citrate, glutamate, fumarate, malate, and aspartate measured by GC-MS in tumours and normal lung from *MYC+N1ICD* and control mice, respectively, given two bolus injections of [U-^{13}C]-glutamine. Data are shown as box and whisker plots where whiskers represent the min and max. **A**: *MYC+N1ICD*, n=7; NC, n=7; **B**: *MYC+N1ICD*, n=4; NC, n=5. * - P <0.05 and ** - P <0.01. *MYC+N1ICD* tumors were compared to control mice using the Welch t-test. See **Table S19**.

Figure S12. SCC tumors utilize glucose-derived carbon into variety of pathways that would produce biosynthetic precursors, reducing equivalents, and protection from ROS. Related to Figures 3-5.

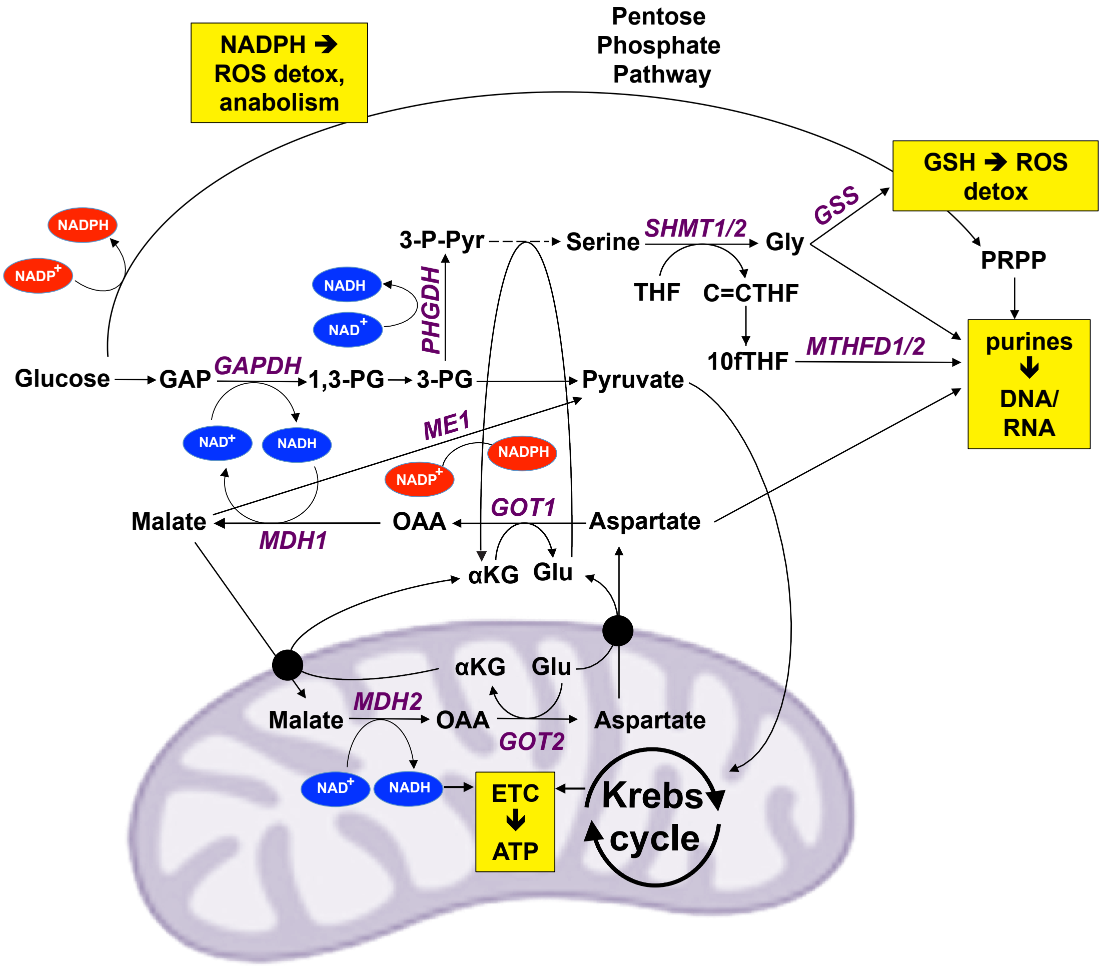

We demonstrate that SCC tumors produce more ^{13}C-lactate from ^{13}C-glucose and shuttle the glucose carbon into a variety of pathways including nucleotide and amino acid biosynthesis. SCC signature genes are shown in purple. The result of flux through these pathways could lead to the biosynthesis of glutathione for ROS protection, NADPH for anabolic process and glutathione reduction, cytosolic NAD^+ to maintain high flux through glycolysis, and mitochondrial NADH that could support the electron transport chain (ETC) and ATP production. ROS- reactive oxygen species; GAP – glyceraldehyde-3-phosphate; 1,3-PG – 1,3-bisphosphoglycerate; 3-PG – 3-phosphoglycerate; 3-P-Pyr- 3-phosphopyruvate; OAA- oxaloacetate; αKG - α-ketoglutarate; Glu – glutamate; THF – tetrahydrofolate; 10fTHF-10-formyltetrahydrofolate.
